# Three New Multifunctional Supramolecular Compounds Based on Keggin-Type Polyoxoanions and 3,5-di(1H-Imidazol-1-yl)benzoic Acid: Syntheses, Structures, and Properties

**DOI:** 10.3390/molecules30030580

**Published:** 2025-01-28

**Authors:** Yanxin Lv, Yang Lu, Xingyuan Yu, Liying Yu, Xiaoshu Qu, Yanyan Yang, Hua Jin, Qingling Wei, Xuemei Li, Xiao-Yang Yu

**Affiliations:** 1 College of Chemical and Pharmaceutical Engineering, Jilin Institute of Chemical Technology, Jilin 132022, China; lvyanxin0204@163.com (Y.L.);; 2 Petrochina Jilin Petrochemical Company Research Institute, Jilin 132022, China

**Keywords:** polyoxometalates, supramolecular structure, photocatalytic property, electrocatalytic property, Hirshfeld surface analysis

## Abstract

Three new inorganic-organic hybrid compounds, (H_3_DIBA)_2_·SiMo_12_O_40_·H_2_O (**1**), (H_3_DIBA)_2_·SiW_12_O_40_·H_2_O (**2**), and [(H_2_DIBA)(H_3_DIBA)]·PMo_12_O_40_·2H_2_O (**3**) (HDIBA = 3,5-di(1H-imidazol-1-yl)benzoic acid) were synthesized by the hydrothermal method. The structures were characterized by single-crystal X-ray diffraction, elemental analysis, powder X-ray diffraction, infrared spectroscopy, and thermogravimetric analysis. These three compounds are all 3D supramolecular structures formed by Keggin-type polyoxometalate anions and HDIBA through intermolecular weak interactions, which have been studied via Hirshfeld surface analysis. The electrochemistry properties of **1** and **3** have been studied, including cyclic voltammetric behaviors and electrocatalytic properties. The study on the removal of organic dye pollutants in water showed that compounds **1** and **3** had an adsorption effect on cationic dye RhB, and the adsorption process conforms to the Langmuir isotherm model. Compound **2** can photocatalytically degrade cationic organic dyes RhB, MB, and CV, and the photocatalytic mechanism study indicates that h^+^ plays a major role in the photocatalytic process.

## 1. Introduction

In recent decades, environmental pollution has increased extensively due to the rapid development of human activities, along with the growth in modern industry and agriculture [1,2,3,4]. Pollutants in water, such as dyes, toxic or corrosive waste acids, and heavy metal ions, not only affect human health but also impact the health of other organisms [5,6,7]. Therefore, it is urgent to adopt appropriate methods and materials to alleviate toxic pollutants in water.

Polyoxometalates (POMs) are a class of metal-oxide clusters with high thermal stability, high negative charge, and remarkable redox properties, which can be used as multielectronic green catalysts with multiple redox-active metal sites and have potential applications in the fields of electrocatalysis, photocatalysis, and catalytic organic reactions [8,9,10]. Since the weak conductivity and high water solubility of POMs limit their applications, it is of great significance to modify and functionalize pure inorganic POMs by covalent or non-covalent bonds in order to modulate the physical and chemical properties of POMs for further practical applications [11,12]. The incorporation of organic ligands (Ls) into the POM system to construct POM-based inorganic-organic hybrid materials has garnered significant research interest [13,14,15]. These Ls can either be directly bonded to POMs (POM-L) [16,17,18] or linked to POMs via metal ions through covalent bonds (POM-M-L) [19,20,21]. Additionally, POMs can serve as counteranions to interact with Ls through non-covalent interactions, leading to the formation of POM-based supramolecular compounds [22,23,24,25]. In contrast to the extensive research on POM-L and POM-M-L complexes formed through covalent interactions, there is a relative scarcity and superficiality in studies focusing on the supramolecular compounds assembled by POM and Ls via non-covalent interactions, such as hydrogen bonding, π···π stacking, van der Waals interactions, and so on [24]. Among these non-covalent interactions, hydrogen bonding and π···π stacking interactions are stronger, and they play important roles in the assembly of supramolecular structures. Hydrogen bonding is the interaction between the protonated hydrogen atom (produced by the donor D-H) and the higher electron density region (:A) of the acceptor. It is directional and selective and may control short-range packing [23,26]. At the supramolecular level, when there is interaction between aromatic rings, π···π stacking is formed, including stacking arrangements (face-to-face, fully aligned, offset, slipped, parallel shifted) and T-shaped conformations with edge or point facing the plane. The number of aromatic moieties, as well as their spatial arrangement, are important factors in determining the extended structural dimension [23]. In fact, these non-covalent interactions can not only affect the structure of supramolecular compounds (such as supramolecular chains, supramolecular layers, and 3D supramolecular network structures) but also affect their properties, making them have potential applications in the fields of catalysis, electricity, and material science and technology [26,27].

Keggin-type polyoxometalates are the most representative compounds in the polyoxometalate family. Keggin-type POMs have the advantages of good hydrophilicity, a simple manufacturing process, and low toxicity [28,29,30]. Most importantly, they can carry out multi-electron redox reactions without changing the original structure. Their reduced and oxidized states can exist stably at the same time, and because of their negative charge, they are easier to combine with other materials to form new functional materials [31].

In the field of crystal design and engineering, there are many influencing factors in the supramolecular assembly process based on POM-organic hybrids, including the type of polyoxometalate, pH level, and the length and rigidity/flexibility of the organic ligands. Among these factors, the selection of appropriate organic ligands is particularly critical, which plays a key role in rationalizing, controlling, and regulating the final supramolecular structures [32,33,34]. Rigid aromatic nitrogen heterocyclic ligands are often used to form POM-based supramolecular compounds, such as imidazole [15], pyridine [17], phenanthroline [14], etc., while the use of nitrogen heterocyclic carboxylic acid ligands has been rarely reported [35]. The tripodal ligand HDIBA (HDIBA = 3,5-di(1H-imidazol-1-yl)benzoic acid), utilizing its imidazole group, benzene ring, and carboxylic acid group to form hydrogen bonding interactions and π···π stacking in structure, is a promising organic ligand candidate for constructing supramolecular structures with Keggin-type polyacids.

In this paper, we successfully synthesized three new inorganic-organic supramolecular compounds by using HDIBA, namely (H_3_DIBA)_2_·SiMo_12_O_40_·H_2_O (**1**), (H_3_DIBA)_2_·SiW_12_O_40_·H_2_O (**2**), and [(H_2_DIBA)(H_3_DIBA)]·PMo_12_O_40_·2H_2_O (**3**) (HDIBA = 3,5-di(1H-imidazol-1-yl)benzoic acid). The crystal structures of these three compounds were characterized, and their electrochemical properties and removal behavior of organic dye pollutants were studied. Furthermore, the Hirshfeld surface analyses were also calculated.

## 2. Results and Discussion

### 2.1. Synthesis Discussion

In the process of hydrothermal synthesis, there are many factors affecting the nucleation and crystal growth of the final product, such as the type and concentration of reactants, the pH value of the reaction system, as well as the reaction time and temperature [32]. The pH value plays a significant role in the formation of compounds. In highly acidic reaction solutions, HIDBA readily becomes protonated. Consequently, it can not only act as a counterion for POM anions but also significantly influence the formation of supramolecular structures. At the same time, it also prevents metal ions from coordinating with the protonated organic ligands. We attempted to synthesize the products by adding only POMs (or the precursors) and the organic ligand during the synthesis process without metal salts, only resulting in powdery mixtures and not suitable for testing. When the pH increases and the acidity decreases, there is a chance to obtain POMs-based compounds [33].

### 2.2. Description of Crystal Structures

Compounds **1**–**3** contain Keggin-type polyoxoanion units (SiMo_12_O_40_^4−^ for **1**, SiW_12_O_40_^4−^ for **2,** and PMo_12_O_40_^3−^ for **3**), and the bond lengths of Si-O, P-O, Mo-O, and W-O are all in the normal ranges [13]. Bond valence sum calculations indicate that W and Mo atoms in these compounds are all in the oxidation states of +VI [29].

#### 2.2.1. Crystal Structures of Compounds **1** and **2**

**Figure 1:**
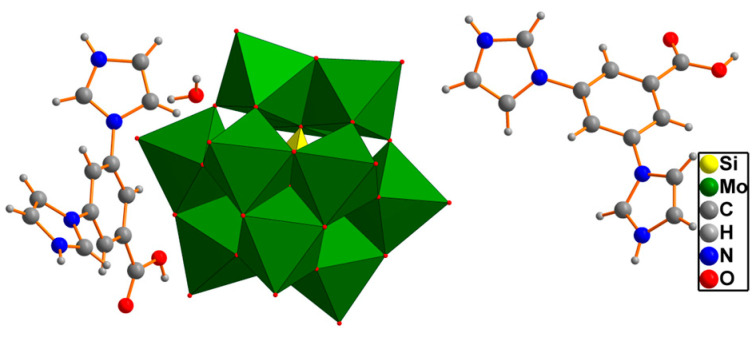
Asymmetric unit of compound **1**.

Single-crystal X-ray diffraction shows that compounds **1** and **2** are isomorphic. Therefore, compound **1** is taken as an example to describe their structures. Compound **1** contains one [SiMo_12_O_40_]^4−^, two protonated H_3_DIBA^2+^ and one lattice water molecule (Figure 1).

**Figure 2:**
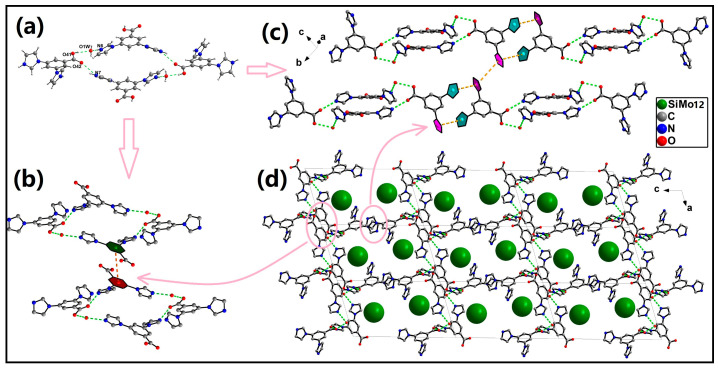
(**a**) Four H_3_DIBA^2+^ and two lattice waters form a ring structure through hydrogen bonding interactions. (**b**) π···π interactions between benzene rings. (**c**) π···π interactions between imidazole rings. (**d**) 3D supramolecular structure of compound **1** (green balls represent the [SiMo_12_O_40_]^4−^ anions).

As the acceptor of the hydrogen bond, O1W hydrogen bonds with imidazole nitrogen N6 of one H_3_DIBA^2+^ and carboxyl oxygen O41 of another H_3_DIBA^2+^ (N6-H6···O1W, 3.042(7) Å, 173°; O41-H41···O1W, 2.729(4) Å, 173°). Imidazole nitrogen N7 acts as the donor of a hydrogen bond, hydrogen bonding to the carboxyl oxygen O42 of an adjacent H_3_DIBA^2+^ (N7-H7A···O42, 2.849(6) Å, 138°). In this way, four H_3_DIBA^2+^ and two lattice waters form a ring structure through hydrogen bonding interactions (Figure 2a). There are π···π interactions between two adjacent rings, including the π···π interactions between benzene rings (centroid to centroid distance of 4.856 Å) and the π···π interactions between imidazole rings (centroid to centroid distances of 4.957 Å and 4.894 Å) (Figure 2b,c). H_3_DIBA^2+^ is connected into a 3D supramolecular network structure via hydrogen bonding and π···π interactions, featuring continuous channels extending along the *b*-axis. The [SiMo_12_O_40_]^4−^ anions fill the channels through hydrogen bonding interactions (Figure 2d).

#### 2.2.2. Crystal Structure of Compound **3**

**Figure 3:**
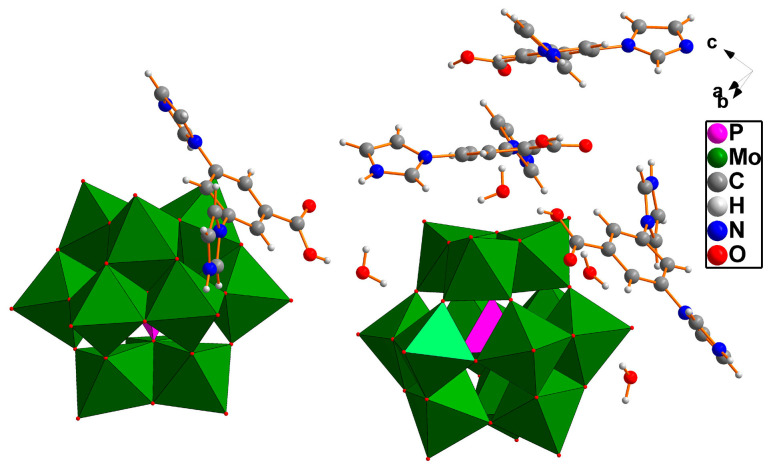
Asymmetric unit of compound **3**.

Single-crystal X-ray diffraction studies revealed that the compound **3** contains two [PMo_12_O_40_]^3−^ molecules, two H_2_DIBA^+^, two H_3_DIBA^2+^, and four lattice water molecules (Figure 3).

**Figure 4:**
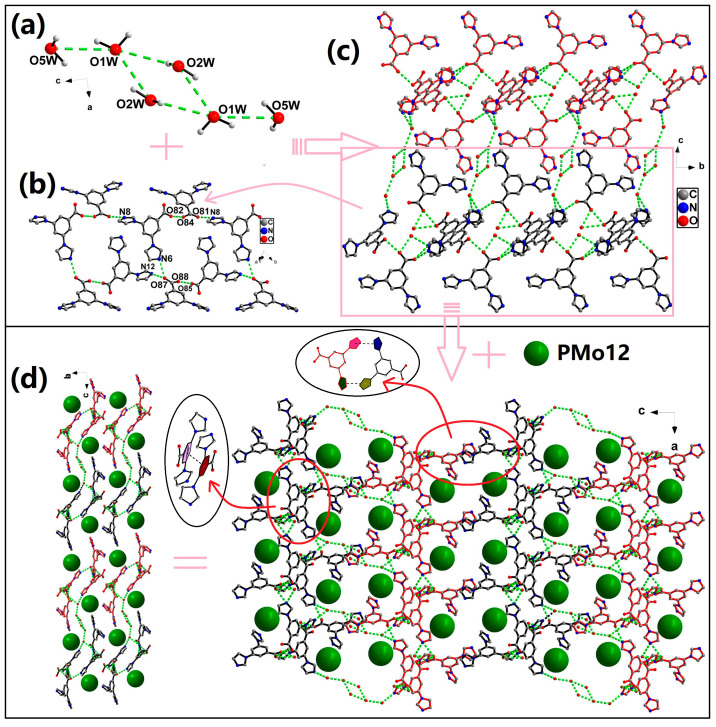
(**a**) The hexanuclear water cluster. (**b**) The organic ligands form 1D supramolecular chains through the hydrogen bond interactions. (**c**) Two adjacent supramolecular chains are linked into a 1D supramolecular band by hexanuclear water clusters through hydrogen bond interactions. (**d**) 3D supramolecular structure of compound **3** (green balls represent the [PMo_12_O_40_]^3−^ anions).

As shown in Figure 4a, four adjacent crystal water molecules are connected through the hydrogen bonding interactions present between them, forming a tetranuclear water cluster (O2W-H2WA···O1W, 2.789(9)Å, 112°; O2W-H2WB···O1W, 2.638(11)Å, 148°; O1W-H1WB···O5W, 2.891(8)Å, 126°). The organic ligands form 1D supramolecular chains through the hydrogen bond interactions between carboxyl and imidazole nitrogen and between carboxyl groups, and two adjacent supramolecular chains are linked into a wider 1D supramolecular band by hexanuclear water clusters through hydrogen bonding interactions, as listed in Table S2 (Figure 4b,c). The neighboring supramolecular bands are connected by the π···π interactions between benzene rings (centroid to centroid distances of 4.814 Å) and the π···π interactions between imidazole rings (centroid to centroid distances of 4.519 Å) to form a 3D supramolecular network structure, with channels extending along the *b*-axis. [PMo_12_O_40_]^3−^ anions fill the channels through hydrogen bonding interactions (Figure 4d).

### 2.3. Hirshfeld Surface Analysis

**Figure 5:**
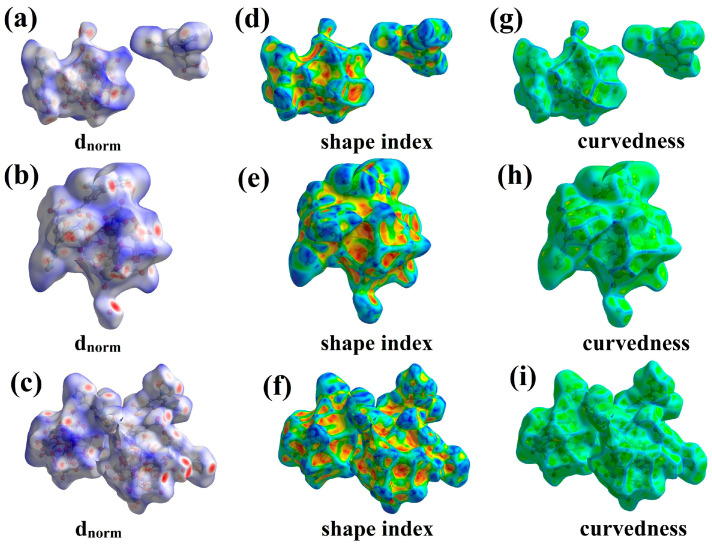
(**a**–**c**) are dnorm of compounds **1**–**3** based on de and di; (**d**–**f**) are the mapping of HS of compounds **1**–**3** on the shape index; (**g**–**i**) are the mappings of HS of compounds **1**–**3** on the curvature.

**Figure 6:**
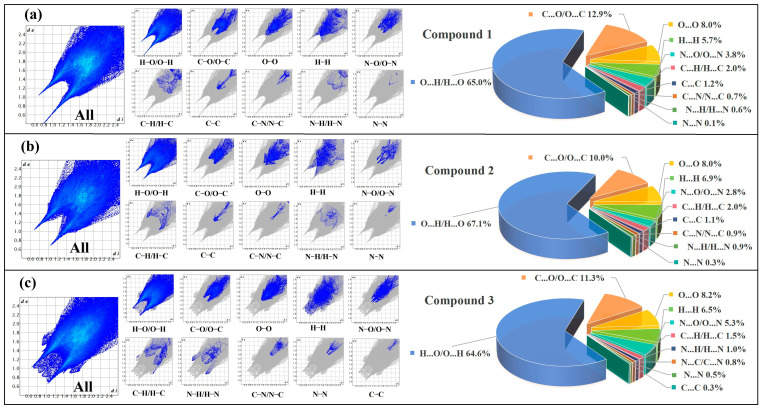
The two-dimensional fingerprint (left) and supramolecular forces contribution ratio (right) of compounds **1** (**a**), **2** (**b**), and **3** (**c**), respectively.

The Hirshfeld surfaces and fingerprint plots were generated based on the structures of the three compounds [22]. The analyses help to understand the geometry and strength of intermolecular interactions through their visualization using appropriate color codes on the Hirshfeld surface, which is defined by points where the contribution of the molecule of interest to the overall electron density is equal to the input from all other molecules [36]. As shown in Figure 5a–c, dnorm is the normalized contact distance based on both de and di (de represents the distance from the point to the nearest nucleus external to the surface, and di is the distance from the internal to the surface). The red, white, and blue regions in the dnorm graph correspond to distances that are less than, equal to, or greater than the sum of the van der Waals radii of the relevant atoms, respectively [37]. In Figure 5d–f, the shape index HS characterizes the intermolecular π···π interactions in compounds **1**–**3**, displaying symmetrical red and blue triangles [37]. Curvature is a mapping of HS on curvature (Figure 5g–i), with the planar regions in the molecule reflected in the curvature map [38]. Two-dimensional fingerprint is an important complement to HS analysis, which can be quantified [39]. As shown in Figure 6, for compounds **1**–**3**, the major contributors to the crystal packing are interactions H···O/O···H, C···O/O···C and O···O, followed by H···H and N···O/O···N. The remaining contacts are less.

### 2.4. PXRD and IR Spectra

In order to check the phase purity of the three compounds, the original samples were tested by powder X-ray diffraction (PXRD) at room temperature. As shown in Figure S1, the measured PXRD spectra are consistent with the simulated spectra of the single crystal data of the three compounds, which proves the purity of the three samples.

The IR spectra of **1**–**3** and HDIBA are shown in Figure S2. The characteristic bands at 1070, 960, 877, and 800 cm^−1^ for **1** are assigned to *v*(Si-O), *v*(Mo-O_d_), *v*(Mo-O_c_-Mo), and *v*(Mo-O_d_-Mo); 1070, 966, 926, and 793 cm^−1^ for **2** are assigned to *v*(Si-O), *v*(W-O_d_), *v*(W-O_c_-W), and *v*(W-O_d_-W); and 1055, 955, 854, and 766 cm^−1^ for **3** are assigned to *v*(P-O), *v*(Mo-O_d_), *v*(Mo-O_c_-Mo), and *v*(Mo-O_d_-Mo), respectively [28,31]. Compared with the spectra of HDIBA, the bands observed at 1720, 1620, and 1540 cm^−1^ for **1** (1700, 1600, and 1540 cm^−1^ for **2**; and 1703, 1606, and 1534 cm^−1^ for **3**) should be attributed to the carboxyl group of the organic ligand. The presence of peaks at 1720, 1700, and 1703 cm^−1^ for these three compounds, respectively, indicates that the carboxyl groups are unprotonated [40]. Additionally, the broad peaks around 3400 cm^−1^ can be attributed to the *v*(O-H) stretching vibration of the crystal water molecules [38], and the occurrence of characteristic vibrations at about 3140 cm^−1^ may be attributed to the *v*(N-H) bond confirming the presence of protonated imidazole groups for **1**–**3** [41].

### 2.5. TG Analyses

As shown in Figure S3, the thermal decomposition processes of **1** and **3** both display three-step weight loss. The first weight loss steps should be due to the loss of crystal water molecules (**1**, 25–115 °C, found, 0.79%, calc., 0.77%; **3**, 25–136 °C, found, 1.59%, calc., 1.52%). The second weight loss step corresponds to losing the organic ligands (**1**, 327–577 °C, found, 25.06%, calc., 22.55%; **3**, 311–554 °C, found, 22.18%, calc., 23.09%). The mass of compounds **1** and **3** did not change until they lost weight again at about 780 °C. In the thermal decomposition processes, compound **1** corresponds to the heat-absorption peaks on the DSC curves appearing at 67, 360, 385, 490, and 552 °C, and compound **3** corresponds to the heat-absorption peaks on the DSC curves appearing at 64, 371, and 459 °C, respectively. The exothermic peak at about 780 °C for both compounds should be attributed to the melting point of MoO_3_ [3]. Compound **2** shows two-step weight loss. The first weight loss step corresponds to the loss of crystal water molecules (25–127 °C, found, 0.91%, calc., 0.53%). The second weight loss step corresponds to losing the organic ligands (352–642 °C, found. 16.94%, calc. 15.51%). Thereafter, until the end of the test, the mass of **2** did not change. Compound **2** corresponds to the heat-absorption peaks on the DSC curves appearing at 52, 510, and 570 °C.

### 2.6. Electrochemical Properties

#### 2.6.1. Cyclic Voltametric Behaviors

**Figure 7:**
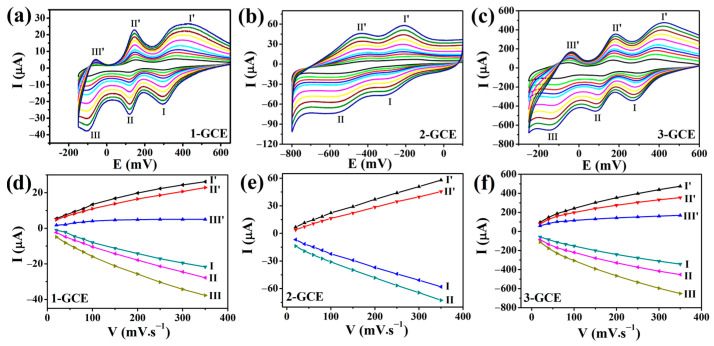
The CV curves of **1**-GCE (**a**), **2**-GCE (**b**), and **3**-GCE (**c**) (scan rates from inner to outer: 20, 40, 60, 80, 100, 120, 140, 160, 180, 200, 250, 300, and 350 mV·s^−1^). The relationship between the peak currents and the scan rates of **1**-GCE (**d**), **2**-GCE (**e**), and **3**-GCE (**f**), respectively.

The electrochemical behaviors of the three compounds have been studied in detail. The cyclic voltammograms of **1**- to **3**-GCEs at different scan rates are shown in Figure 7 (**1**- and **3**-GCEs in 0.1 M H_2_SO_4_ + 0.5 M Na_2_SO_4_ aqueous solution, **2**-GCE in 0.5 M Na_2_SO_4_ aqueous solution).

In the potential range of −150 mV~+650 mV for **1**-GCE and −250~+600 mV for **3**-GCE, there were three reversible redox peaks with the half-wave potentials (E_1/2_ = (Epc + Epa)/2) of +337 mV (I–I’), 148 mV (II–II’), and −77 mV (III–III’) for **1**-GCE; and 336 mV (I–I’), 136 mV (II–II’), and −85 mV (III–III’) for **3**-GCE (scan rate: 100 mV·s^−1^), respectively, corresponding to the three consecutive two-electron redox processes of [XMo_12_O_40_]^n−^ (X = Si, n = 4 for **1**; X = P, n = 3 for **3**). The three consecutive two-electron redox processes of the two compounds can both be expressed as follows [42]:

XMo_12_^VI^O_40_^n−^ + 2H^+^ + 2e^−^ → H_2_XMo_10_^VI^Mo_2_^V^O_40_^n−^, (1)

H_2_XMo_10_^VI^Mo_2_^V^O_40_^n−^ + 2H^+^ + 2e^−^ → H_4_XMo_8_^VI^Mo_4_^V^O_40_^n−^, (2)

H_4_XMo_8_^VI^Mo_4_^V^O_40_^n−^ + 2H^+^ + 2e^−^ → H_6_XMo_6_^VI^Mo_6_^V^O_40_^n−^. (3)

For **2**-GCE, there are two pairs of reversible redox peaks (I–I’ and II–II’) in the potential range of −800~+100 mV, and the half-wave potentials (scan rate: 100 mV·s^−1^) were −254 mV (I–I’) and −488 mV (II–II’), respectively, which should be associated with the two consecutive one-electron redox processes of SiW_12_. The two processes can be expressed as follows [43]:

SiW_12_^VI^O_40_^4−^ + H^+^ + e^−^ → HSiW_11_^VI^W^V^O_40_^4−^, (4)

HSiW_11_^VI^W^V^O_40_^4−^ + H^+^ + e^−^ → H_2_SiW_10_^VI^W_2_^V^O_40_^4−^. (5)

For the three compounds, as the scan rates varied from 20 to 350 mV·s^−1^, the cathodic peak potential gradually shifted to the negative direction, and the corresponding anodic peak potential shifted to the positive direction. The peak currents are directly proportional to the scan rates, indicating that all redox processes are surface-controlled [40].

#### 2.6.2. Electrocatalytic Properties

**Figure 8:**
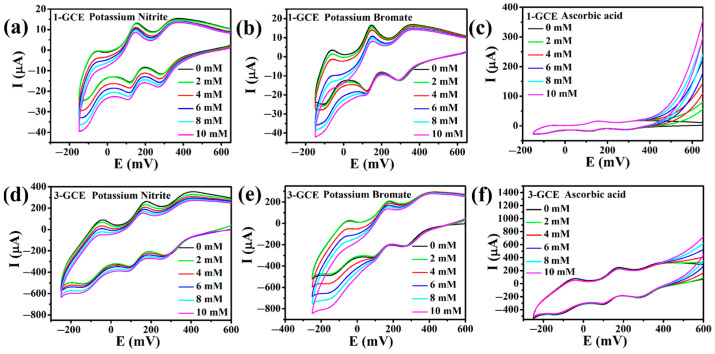
Cyclic voltammograms of **1**-GCE in 0.1 M H_2_SO_4_ + 0.5 M Na_2_SO_4_ aqueous solution containing 0, 2, 4, 6, 8, and 10 mM NO_2_^−^ (**a**), BrO_3_^−^ (**b**), and AA (**c**). Cyclic voltammograms of **3**-GCE in 0.1 M H_2_SO_4_ + 0.5 M Na_2_SO_4_ aqueous solution containing 0, 2, 4, 6, 8, and 10 mM NO_2_^−^ (**d**), BrO_3_^−^ (**e**), and AA (**f**).

The study on the electrocatalytic performances of **1**- and **3**-GCEs reveals that their electrocatalytic characteristics are comparable. As shown in Figure 8a,d, as the NO_2_^−^ concentration in the solution increases, the reduction peak currents for I–I’, II–II’, and III–III’ gradually increase, while the corresponding oxidation peak currents gradually decrease [44]. The phenomenon indicates that **1**- and **3**-GCEs have electrocatalytic activity for the reduction of NO_2_^−^. And the proposed reaction mechanism toward NO_2_^−^ can probably be described by the following equations [42,44]: H_2_XMo_10_^VI^Mo_2_^V^O_40_^n−^ + 2NO_2_^−^ → XMo_12_O_40_^n−^ + products (containing N); H_4_XMo_8_^VI^Mo_4_^V^O_40_^n−^ + 4NO_2_^−^ → XMo_12_O_40_^n−^ + products (containing N); H_6_XMo_6_^VI^Mo_6_^V^O_40_^n−^ + 6NO_2_^−^ → XMo_12_O_40_^n−^ + products (containing N). However, with the increase of the concentration of BrO_3_^−^, only the reduction peak currents of the III–III’ peak increased, and the corresponding oxidation peak currents gradually decreased (Figure 8b,e), while the other two reduction peak current peaks did not change significantly. The result indicates that **1**- and **3**-GCEs also have electrocatalytic activity for the reduction of BrO_3_^−^. And the reaction mechanism toward BrO_3_^−^ can probably be described by the following equations [45]: H_6_XMo_6_^VI^Mo_6_^V^O_40_^n−^ + BrO_3_^−^ → XMo_12_O_40_^n−^ + Br^−^ + 3H_2_O.

We also studied their electrocatalytic activities for the oxidation of ascorbic acid (AA; formula, C_6_H_8_O_6_). With the addition of AA, the oxidation peak currents of **1**- and **3**-GCEs progressively increase in the potential range, while the relevant reduction peak currents gradually decrease (Figure 8c,f). The results indicate that **1**- and **3**-GCEs are electro-catalysts for the oxidation of AA. The probable oxidation mechanism may be as follows [44]: XMo_12_^VI^O_40_^n−^ + C_6_H_8_O_6_ → H_2_XMo_10_^VI^Mo_2_^V^O_40_^n−^ + C_6_H_6_O_6_. The results of the electrocatalytic tests indicate that **1**- and **3**-GCEs have bifunctional electrocatalytic activities for the reduction of NO_2_^−^ and BrO_3_^−^ and the oxidation of AA.

**Figure 9:**
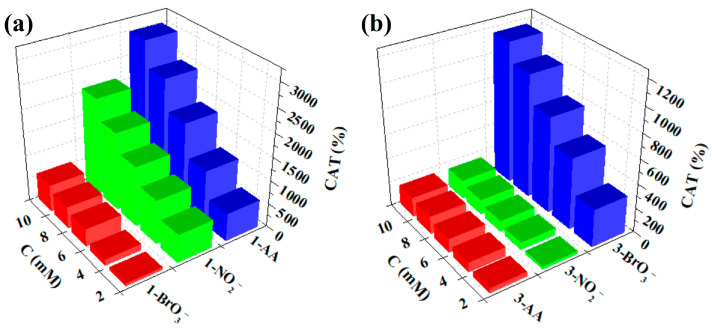
Histogram of the CATs vs. the concentrations of NO_2_^−^, BrO_3_^−^, and AA for **1**-GCE (**a**) and **3**-GCE (**b**).

Depending on the changes in peak intensity with the addition of BrO_3_^−^, NO_2_^−^, and AA, the electrocatalytic efficiencies (CATs) of **1**- and **3**-GCEs were calculated using the following formula: CAT = 100% × [Ip(C, substrate) − Ip(C)]/Ip(C), in which Ip(C, substrate) and Ip(C) are defined as the anodic peak current intensities with and without NO_2_^−^ (or BrO_3_^−^, or AA), respectively. As shown in Figure 9, comparison shows that the CATs of **3**-GCE for BrO_3_^−^ are better than those of **1**-GCE, while the CATs of **1**-GCE for NO_2_^−^ and AA are higher than those of **3**-GCE, which may be due to the structural differences.

### 2.7. Performance in Removing Organic Dyes

POM anions have oxygen-rich surfaces, high negative charges, and a large number of potential active sites for efficient use of light energy on the surface, which are beneficial for photocatalytic degradation or adsorptive removal of harmful organic dyes [8]. Up to now, it has been reported that polyoxometalates can be used as excellent materials for removing organic dye pollutants [15].

We investigated the performance of the three compounds for photocatalytic degradation or adsorption removal of organic dyes. The results show that compound **2** exhibited significant photocatalytic degradation of dyes, while compounds **1** and **3** were more effective in the adsorption of organic dyes.

#### 2.7.1. Photocatalytic Properties for Organic Dyes

**Figure 10:**
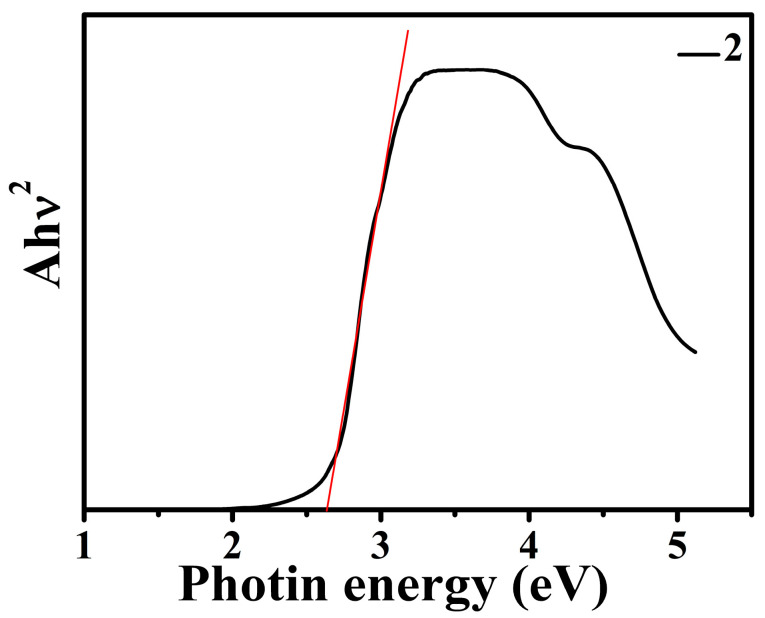
Band gap energy of **2** according to the Kubelka–Munk formula.

The diffuse reflectance spectrum of compound **2** was tested to obtain its band gap energy (Eg). As shown in Figure 10, the absorption data were calculated from the reflection by the Kubelka–Munk function F against E, and the energy gap (Eg) extrapolated from the linear part of the absorption edge was 2.63 eV. Band gap calculation indicated that compound **2** has semiconductor properties and can be used as a potential photocatalytic material.

**Figure 11:**
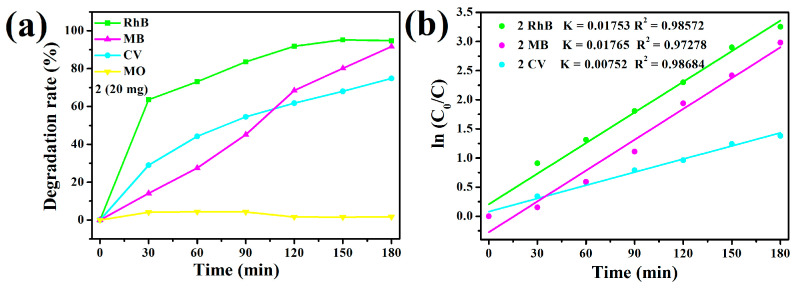
(**a**) Degradation rates of RhB (40 ppm), MB (40ppm), CV (20 ppm) and MO (40 ppm) after 180 min under 300 W Xe light using compound **2** as a catalyst (20 mg). (**b**) The linear plots of the pseudo-first-order kinetics of RhB, MB, and CV.

Using compound **2** as a photocatalyst, as shown in Figure 11a, after 180 min, the photocatalytic degradation rates of the cationic dyes RhB, MB, and CV solutions were about 94.78%, 91.65%, and 74.83%, respectively, while the anionic dye MO was almost not degraded (Figure S4). After 24 h, all the photocatalytic degradation rates did not change significantly. As shown in Figure S5, the degradation efficiency did not decrease after five cycles. This demonstrates that compound **2** has good recoverability and reutilization for the three cationic dyes. The degradation rate constants for RhB, MB, and CV were calculated using the ln(C_0_/C) versus time curves, and the results showed that the degradation processes of the three cationic dyes by compound **2** conform to pseudo-first-order kinetics [29] (Figure 11b).

**Figure 12:**
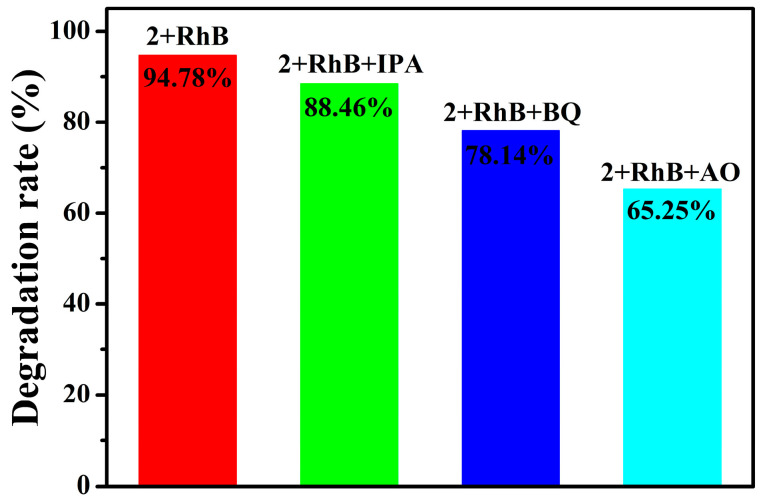
Photocatalytic degradation of the RhB solution (40 ppm) with different scavenger solutions for **2** (20 mg).

In order to study the photocatalytic mechanism of compound **2**, capture experiments were conducted to determine the main active species. Ammonium oxalate (AO), isopropanol (IPA), and benzoquinone (BQ) were used to scavenge photogenerated holes, hydroxyl radicals, and superoxide radicals, respectively. The photocatalytic degradation of RhB dye (40 ppm) was taken as an example. As shown in Figure 12, when AO was added, the photocatalytic degradation rate decreased significantly, indicating that h^+^ plays a major role in the photocatalytic process. The possible mechanism should be described by the following equations [45]:

POMs + hv → *POMs + h^+^, (6)

H_2_O + h^+^ → ^∙^OH + H^+^, (7)

dyes + ^∙^OH → degradation products, (8)

dyes + h^+^ → degradation products, (9)

*POMs + O_2_ → POMs + ^∙^O_2_^−^, (10)

dyes + ^∙^O_2_^−^ → degradation products. (11)

#### 2.7.2. Adsorption Properties for Organic Dyes

**Figure 13:**
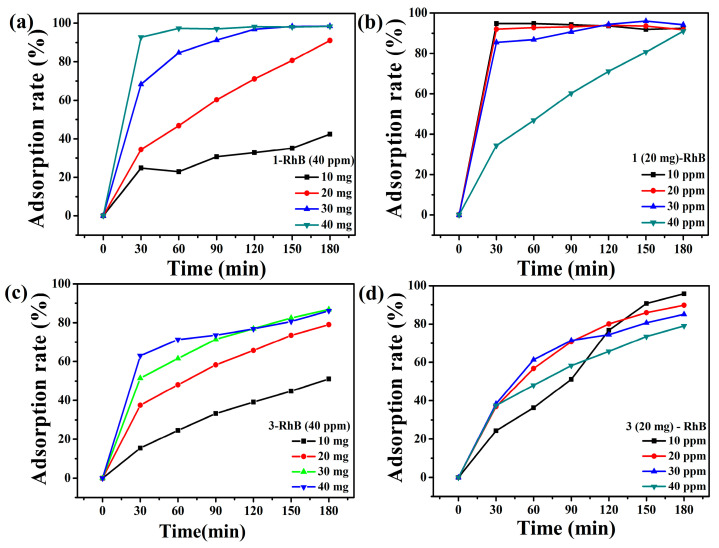
Effect of adsorbent dosages **1** (**a**) and **3** (**c**); RhB dye concentration on adsorption efficiency with **1** (**b**) and **3** (**d**).

Taking the cationic RhB dye as an example, the effects of adsorption dosage and dye concentration on the adsorption degradation efficiency were investigated with compounds **1** and **3** as adsorbents. As shown in Figure 13a,c, the adsorption rates were both increased when the dosages of the adsorbents were increased from 10 mg to 40 mg. When the adsorbent dosage was 40 mg, the optimum adsorption rates were 98.46% (**1**) and 86.82% (**3**) after 180 min, respectively, which may be due to the increase in adsorption sites caused by the increase in adsorbent dosage [46]. By comparing the relationship between the adsorption rates and the dosages of the adsorbents, it can be found that when the dosages are 20 mg, the adsorption rates after 180 min are 91.04% (**1**) and 80.00% (**3**), respectively, which are close to the adsorption rates of 40 mg adsorbents. Therefore, from the perspective of energy conservation, we selected 20 mg of adsorbents to explore the effect of dye concentration on the adsorption rates.

As shown in Figure 13d and Figure S7, for compound **3**, the adsorption rates decreased when the concentration of RhB dye was varied from 10 ppm to 40 ppm. When the dye concentration was 10 ppm, the degradation rate reached the maximum value of 95.72%. This may be due to the saturation of the catalytic sites. For compound **1**, with the increase in dye concentration, the adsorption rates remained almost unchanged at more than 90% (Figure 13b and Figure S6). The adsorption rates of compounds **1** and **3** did not change significantly after 24 h. After five cycles, the adsorption efficiencies did not decrease significantly, indicating the stability of their adsorption (Figure S8). The adsorption effect should be attributed to the strong attraction of POM anions to cationic dyes [47]. The results of the adsorption experiments showed that the adsorption effect of compound **1** on RhB was much better than that of compound **3** within 180 min.

**Figure 14:**
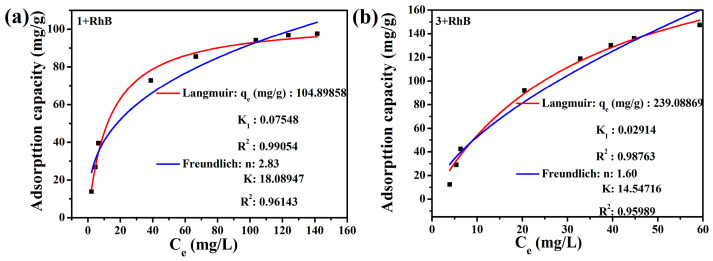
Adsorption isothermal models fitted with Langmuir and Freundlich of **1** (**a**) and **3** (**b**).

The adsorption isotherm was used to understand the mobility or retention rate of the dye between the solid phase and the liquid phase. The Langmuir and Freundlich isotherm models were used to fit the adsorption isotherms. The results showed that the adsorption isotherms were in good agreement with the Langmuir model (Figure 14). The maximum adsorption capacity are 104.90 mg/g for **1** (R^2^ > 0.98) and 239.09 mg/g for **3** (R^2^ > 0.98), indicating that the monolayer adsorption of the dye occurs at a specific homogeneous site [48].

## 3. Experimental Section

### 3.1. Materials and Methods

All the starting materials were obtained commercially and employed without additional purification. The IR spectra (KBr pellets) were recorded using a Perkin-Elmer Spectrum One FT-IR spectrometer in the range of 400–4000 cm^−1^ at room temperature. Thermogravimetric (TG) analyses were tested on a Perkin-Elmer TGA7 instrument with a heating rate of 10 °C·min^−1^ from room temperature to 900 °C in air. Elemental analyses for C, H, and N were determined on a Perkin-Elmer 2400 Elemental Analyzer. Powder X-ray diffraction (PXRD) patterns were collected using a Bluker D8 Advance X-ray diffractometer (graphite monochromatized Cu-Ka, *λ* = 0.15418 Å) in the 2θ range of 5–50° with an increment of 0.02. Electrochemical behaviors were tested on a CHI760E electrochemical workstation. Photocatalytic performance was operated using the CEL-HXF300 AULIGHT, Beijing. UV–Vis spectra were recorded on a TU-1900 spectrophotometer. The UV–Vis diffuse-reflectance spectroscopy (UV–Vis DRS) spectra were characterized using UV–Vis NIR spectrophotometer (Lambda 1050+) with barium sulfate (BaSO_4_) as the standard.

### 3.2. Syntheses

#### 3.2.1. Synthesis of (H_3_DIBA)_2_·SiMo_12_O_40_·H_2_O (**1**)

AgNO_3_ (0.0170 g, 0.10 mmol), Na_2_SiO_3_·9H_2_O (0.0284 g, 0.10 mmol), Na_2_MoO_4_·2H_2_O (0.1210 g, 0.50 mmol), HDIBA (0.0127 g, 0.05 mmol), and 10 mL H_2_O were mixed and stirred for 30 min. The pH value of the mixture was adjusted to 1.80 with 1.0 mol·L^−1^ HNO_3_, and the mixture was further stirred for another 30 min. Subsequently, the mixture was transferred into a 15 mL Teflon-lined autoclave, sealed in an oven, and heated at a constant temperature of 170 °C for 72 h. The mixture was then cooled down to room temperature at a rate of 10 °C/h, yielding the yellow block crystals of compound **1**. Yield 30% (based on Mo). Anal. Calc. for C_26_H_26_Mo_12_N_8_O_45_Si, C: 13.27; H: 1.11; N: 4.77 (%). Found C: 14.01; H: 1.18; N: 4.51 (%). IR (KBr, cm^−1^): 3610 m, 3140 m, 1720 m, 1620 w, 1540 m, 1400 m, 1070 s, 960 s, 877 s, 800 s.

#### 3.2.2. Synthesis of (H_3_DIBA)_2_·SiW_12_O_40_·H_2_O (**2**)

Zn(CH_3_COO)_2_·2H_2_O (0.0548 g, 0.25 mmol), H_4_SiW_12_O_40_·H_2_O (0.1448 g, 0.05 mmol), HDIBA (0.0127 g, 0.05 mmol), and 10 mL H_2_O were mixed and stirred for 30 min. The mixture was adjusted to a pH of approximately 1.90 with 1.0 mol·L^−1^ HCl, and then further stirred for an additional 30 min. Subsequently, the mixture was poured into a 15 mL Teflon-lined autoclave, sealed, and placed in an oven for heating at a constant temperature of 150 °C for 96 h. Afterward, the mixture was cooled to room temperature at a rate of 10 °C/h, resulting in the formation of white block crystals. Yield 20% (based on W). Anal. Calc. for C_26_H_26_N_8_O_45_SiW_12_ C: 9.16; H: 0.76; N: 3.29 (%). Found C: 9.15; H: 0.74; N: 3.27 (%). IR (KBr, cm^−1^): 3580 w, 3150 m, 1700 m, 1600 m, 1540 s, 1390 m, 1070 m, 966 s, 926 s, 793s.

#### 3.2.3. Synthesis of [(H_2_DIBA)(H_3_DIBA)]·PMo_12_O_40_·2H_2_O (**3**)

The synthesis method of compound **3** is similar to that of **2**. The reactants are Cu(CH_3_COO)_2_·H_2_O (0.0998 g, 0.50 mmol), H_3_PMo_12_O_40_·H_2_O (0.1825 g, 0.10 mmol), and HDIBA (0.0127 g, 0.05 mmol), and the pH of the mixture was 1.15. Black hexagonal prismatic of compound **3** was obtained in 40% yield (based on Mo). Anal. Calc. for C_26_H_27_Mo_12_N_8_O_46_P: C: 13.17; H: 1.14; N: 4.73 (%). Found C: 13.27; H: 1.21; N: 4.67 (%). IR (KBr, cm^−1^): 3461 w, 3130 m, 1703 w, 1606 m, 1534 s, 1386 m, 1055 s, 955 s, 854 s, 766 m.

### 3.3. X-Ray Crystallography

Crystal data for **1**–**3** were collected with Mo Kα radiation (*λ* = 0.71073 Å, graphite monochromator) on a Bruker APEX CCD area detector at 298 K. All structures were solved using direct methods and refined by full-matrix least-squares on *F*^2^ with the SHELXTL program. All non-hydrogen atoms were refined with anisotropic thermal parameters. The hydrogen atoms of the ligands were placed theoretically. The CCDC reference numbers were 2,405,584 (**1**), 2,404,837 (**2**), and 2,404,838 (**3**), respectively. Details of the final refinements are given in Table 1. Selected bond lengths and angles are listed in Table S1. Hydrogen bonding interactions are listed in Table S2.

### 3.4. Preparation of the Working Electrode

Compounds **1**–**3** were used to modify glass carbon electrodes (**1**-, **2**-, and **3**-GCEs) as follows: (1) The glass carbon electrodes (GCEs, 3 mm diameter) were polished on a polishing pad with 0.3 and 0.05 μm alumina powders and then dried with nitrogen stream. (2) 12 mg of each compound was mixed with carbon black in a mass ratio of 1:2 by grinding. And the resulting mixture was dispersed into 200 μL of a 0.5 wt% Nafion solution and subjected to ultrasonication for 30 min to ensure a homogeneous dispersion. (3) Finally, 12 μL of the solution was dropped on the surface of GCE and dried in air before testing. In the process, Nafion solution served as both an adhesive and a protective agent to ensure that the compounds can be tightly modified on the surface of the GCE electrode. The conventional three-electrode system was used: working electrode, auxiliary electrode (platinum wire), and reference electrode (Ag/AgCl).

### 3.5. Organic Dye Removal Experiment

The behavior of the three compounds in removing organic dyes was investigated through photocatalytic degradation and adsorptive removal of the dyes.

The process of the photocatalytic degradation of organic dyes experiment is as follows: Three cationic dyes, Rhodamine B (RhB), Methylene Blue (MB), and Crystal Violet (CV), along with the anionic dye Methyl Orange (MO), were selected to investigate the photocatalytic degradation performance of the photocatalyst on organic dyes. A total of 20.0 mg of the photocatalyst was dispersed in 50.0 mL of the organic dye solution and stirred in the dark for 24 h to reach adsorption and desorption equilibrium. The organic dye solution containing the photocatalyst was then continuously stirred under full-spectrum irradiation from a 300 W Xe lamp. Every 30 min, 1.0 mL of the solution was taken for UV–visible spectral testing.

The process of the experiment for the adsorption removal of organic dyes is as follows: The cationic dye RhB was selected to investigate the adsorption performance of the adsorbent for organic dyes. Under dark conditions, the adsorbent was added to the organic dye solution (50 mL) and continuously stirred, with 1.0 mL samples taken at regular intervals for UV–visible spectroscopy testing.

## 4. Conclusions

In this study, three new Keggin-type polyoxometalate-based inorganic-organic supramolecular compounds were synthesized and characterized. The intermolecular interactions in the compounds were calculated via Hirshfeld surface analysis. The results indicated that the major contributors to the crystal packing are interactions H···O/O···H, C···O/O···C, and O···O. Electrochemical studies showed that compounds **1**–**3** had electrochemical behavior, and **1**-GCE and **3**-GCE have electrocatalytic activity for the reduction of BrO_3_^−^ and NO_2_^−^ and the oxidation of AA. In addition, the study on the removal of organic dye pollutants in water showed that compounds **1** and **3** had adsorption effects on cationic dye RhB, and the adsorption process conforms to the Langmuir isotherm model. Compound **2** can photocatalytically degrade cationic organic dyes RhB, MB, and CV, and trapping experiments showed that h^+^ may be the main active substance for the decomposition of organic dyes in photocatalytic reactions. Other properties for the above compounds are still under development.

## Figures and Tables

**Table 1:** Crystal data and structure refinements for compounds **1**–**3**.

	1	2	3
Formula	C_26_H_26_N_8_O_45_SiMo_12_	C_26_H_26_N_8_O_45_SiW_12_	C_26_H_27_N_8_O_46_PMo_12_
Mol. wt.	2349.92	3404.84	2369.81
Crystal system	triclinic	triclinic	triclinic
Space group	*P* -1	*P* -1	*P* -1
*a* (Å)	12.7558(3)	12.8967(18)	12.9557(4)
*b* (Å)	12.9565(4)	13.121(2)	12.9815(4)
*c* (Å)	19.9055(5)	19.915(3)	38.3126(13)
*α* (°)	94.5200(10)	94.731(6)	83.6830(10)
*β* (°)	102.4780(10)	102.099(5)	87.1470(10)
*γ* (°)	118.0400(10)	118.522(5)	61.1820(10)
*V* (Å^3^)	2772.25(13)	2829.0(8)	5611.3(3)
*Z*	2	2	4
*Dc* (g cm^−3^)	2.815	3.997	2.805
*µ* (mm^−1^)	2.759	24.422	2.736
*F* (000)	2232	3000	4504
Reflection collected	71,691	58,097	139,559
*R*_int_	0.0335	0.0533	0.0365
GOF	1.090	1.048	1.119
Final *R* ^ab^ indices [*I* > 2*σ*(*I*)]	*R*_1_ = 0.0352, *wR*_2_ = 0.0515	*R*_1_ = 0.0648, *wR*_2_ = 0.0906	*R*_1_ = 0.0646, *wR*_2_ = 0.1006
*R* indices (all data)	*R*_1_ = 0.0254, *wR*_2_ = 0.0568	*R*_1_ = 0.0424, *wR*_2_ = 0.1097	*R*_1_ = 0.0497, *wR*_2_ = 0.1086

^a^ *R*_1_ = Σǀǀ*Fo*ǀ − ǀ*Fc*ǀǀ/Σǀ*F*ǀ; ^b^ *wR*_2_ = ǀΣ*w* (ǀ*Fo*ǀ^2^ − ǀ*Fc*ǀ^2^)^2^/Σǀ*w*(*F*)^2^ǀ^1/2^.

## Data Availability

Data are contained within the article and Supplementary Materials.

## Supplementary Materials

The following supporting information can be downloaded at: https://www.mdpi.com/article/10.3390/molecules30030580/s1, Figure S1: The PXRD patterns of compounds **1**–**3**. Figure S2: The IR curves of compounds **1**–**3** and HDIBA. Figure S3: The TG curves of compounds **1**–**3**. Figure S4: Absorption spectra of RhB (a), MB (b), CV (c) and MO (d) solutions under 300 W Xe light with full spectrum in the presence of compound **2**. Figure S5: The photocatalytic cycle experiments of compound **2** on RhB, MB and CV. Figure S6: Adsorption spectra of different RhB dye concentrations and different doses of compound **1**. Figure S7: Adsorption spectra of different RhB dye concentrations and different doses of compound **3**. Figure S8: Adsorption cycle experiments of compounds **1** and **3** on RhB. Table S1: The selected bond lengths (Å) and angles (°) of compounds **1**–**3**. Table S2: Selected hydrogen bonds of compounds **1**–**3**.
