# Supplementary material for: Three New Multifunctional Supramolecular Compounds Based on Keggin-Type Polyoxoanions and 3,5-di(1H-Imidazol-1-yl)benzoic Acid: Syntheses, Structures, and Properties
Source: Molecules. 2025 Jan 28;30(3):580. doi: 10.3390/molecules30030580 (PMC11820590; doi:10.3390/molecules30030580)
Supplement: Supplementary file 1 [file molecules-30-00580-s001.zip › molecules-3400078-supplementary.pdf]

Supplement files

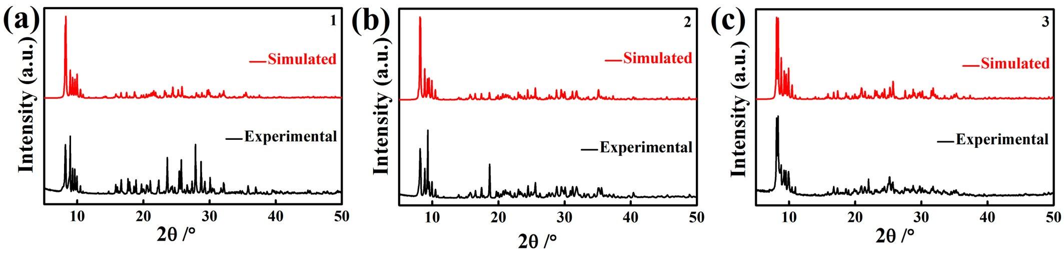

Figure S1. The PXRD patterns of compounds **1-3**.

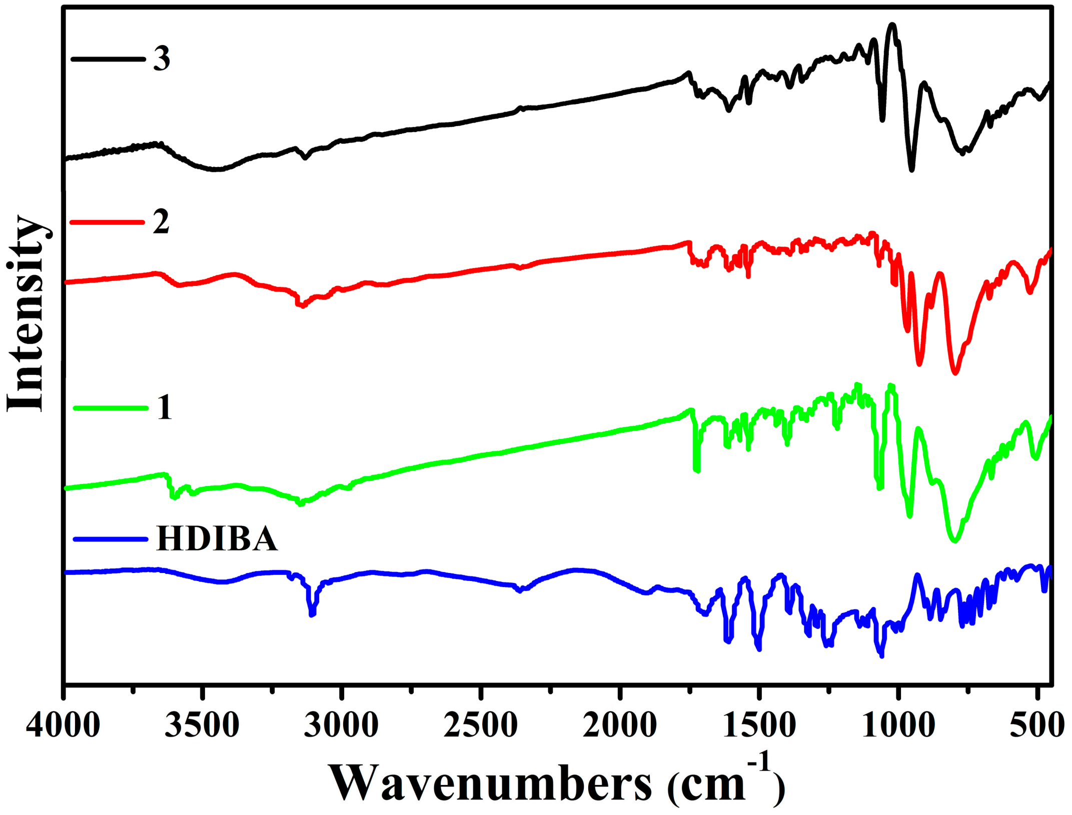

Figure S2. The IR curves of compounds **1-3** and HDIBA.

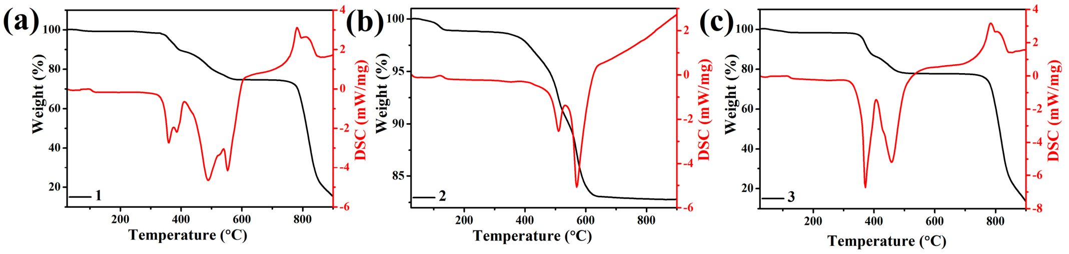

Figure S3. The TG curves of compounds **1-3**.

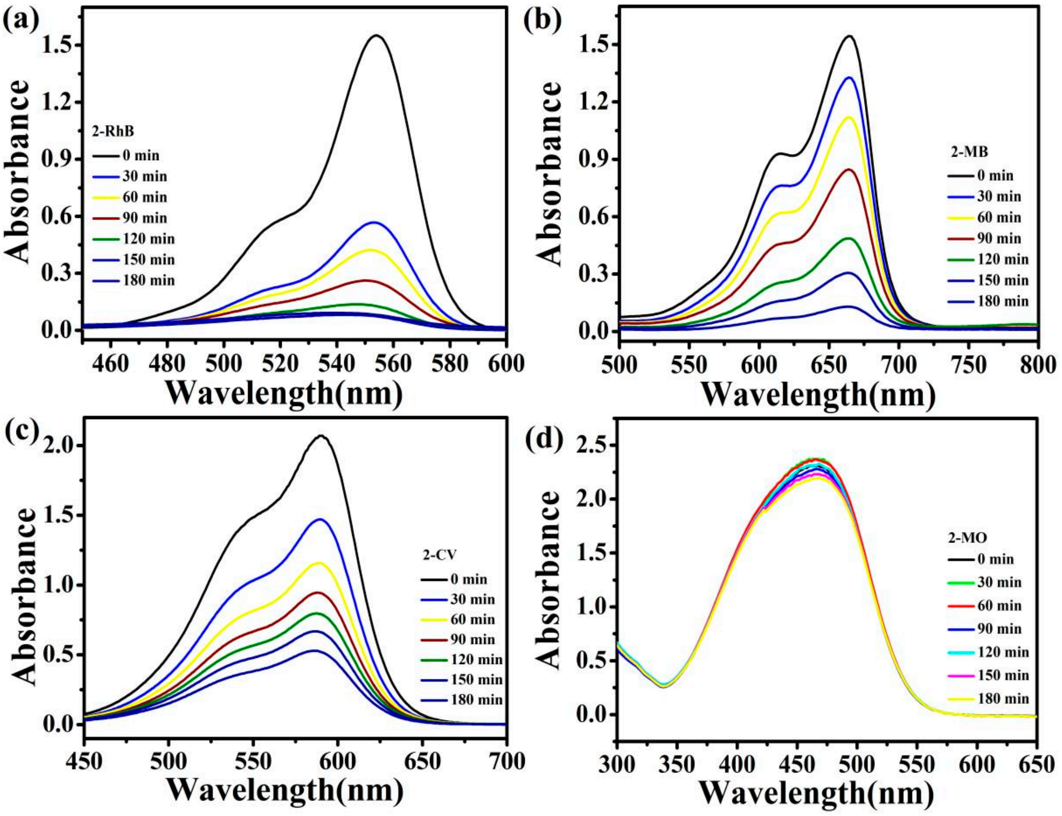

Figure S4. Absorption spectra of RhB (a), MB (b), CV (c), and MO (d) solutions under 300 W Xe light with full spectrum in the presence of compound **2**.

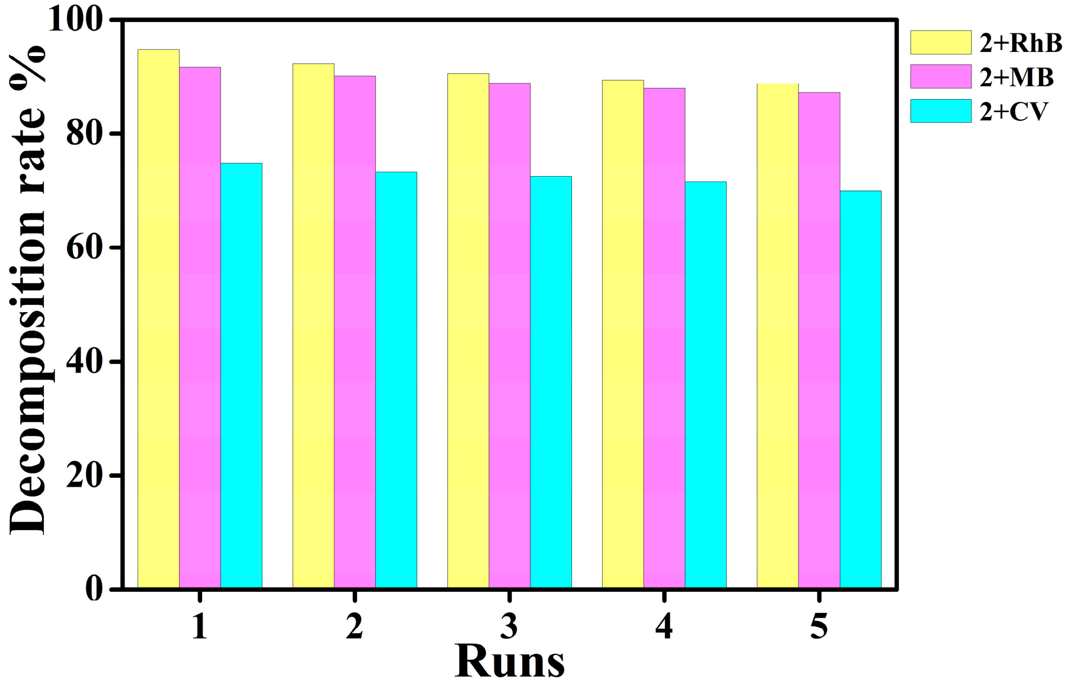

Figure S5. The photocatalytic cycle experiments of compound **2** on RhB, MB, and CV.

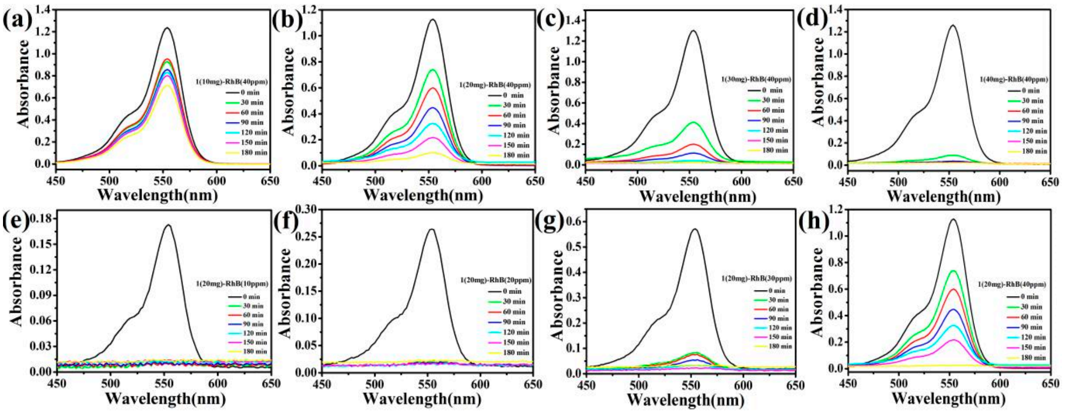

Figure S6. Adsorption spectra of different RhB dye concentrations and different doses of compound **1**.

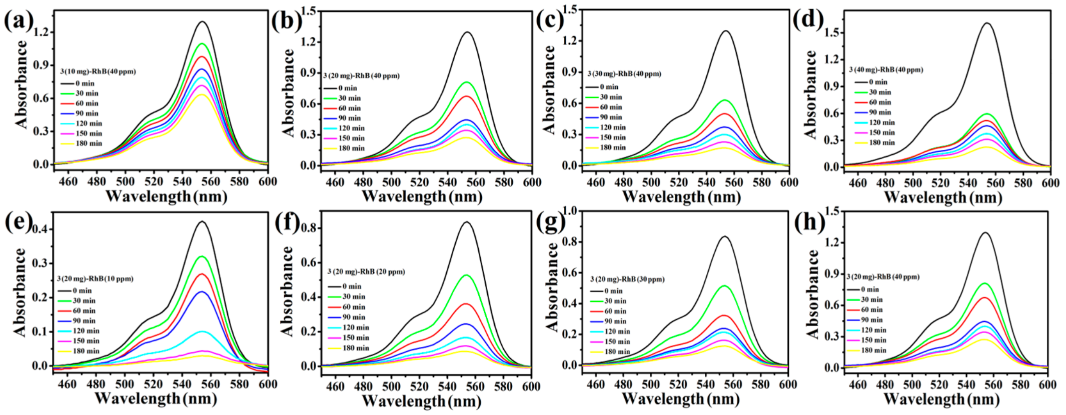

Figure S7. Adsorption spectra of different RhB dye concentrations and different doses of compound **3**.

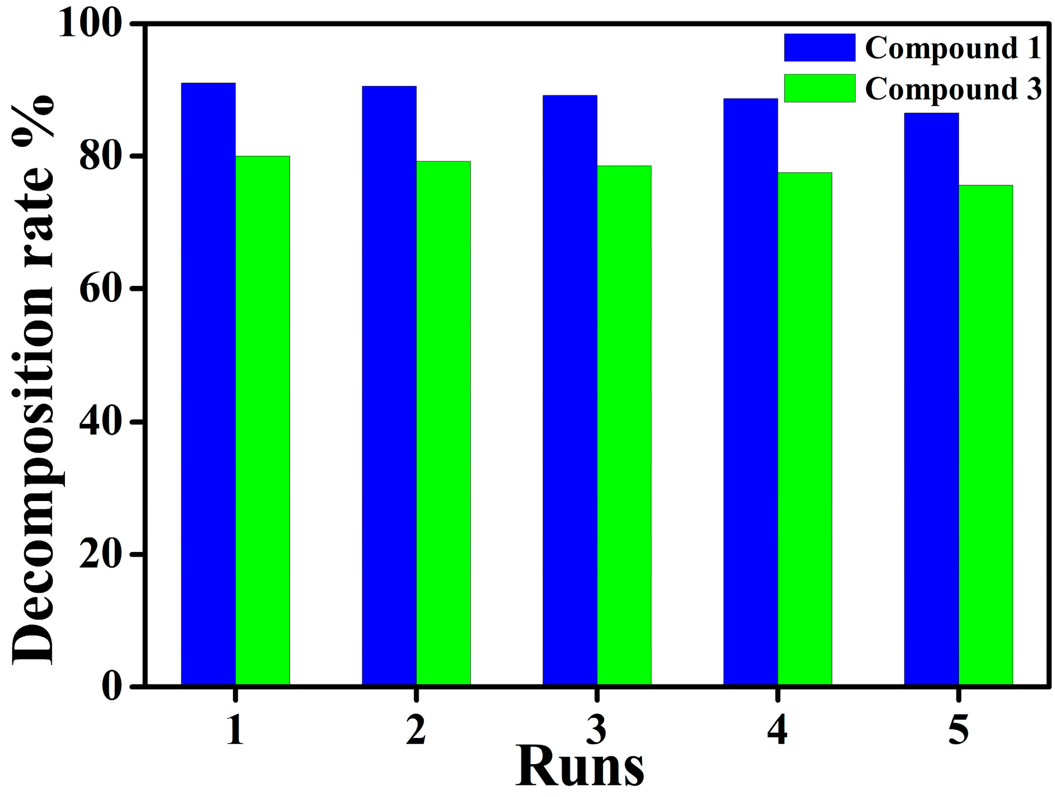

Figure S8. Adsorption cycle experiments of compounds **1** and **3** on RhB.

Table S1. The selected bond lengths (Å) and angles (°) of compounds **1-3**.

**1**			
Si1-O27	1.622(3)	Si1-O29	1.627(2)
Si1-O28	1.629(2)	Si1-O38	1.624(3)
Mo1-O1	1.676(3)	Mo7-O17	1.820(3)
Mo1-O14	2.054(3)	Mo7-O21	1.835(3)
Mo1-O16	1.857(3)	Mo7-O22	2.030(3)
Mo1-O18	1.812(3)	Mo7-O27	2.357(2)
Mo1-O19	1.987(3)	Mo7-O31	2.022(3)
Mo1-O38	2.369(2)	Mo7-O40	1.685(3)
Mo2-O2	1.698(3)	Mo8-O8	1.688(3)
Mo2-O13	1.806(3)	Mo8-O20	1.973(3)
Mo2-O15	2.036(3)	Mo8-O22	1.811(3)
Mo2-O16	1.998(3)	Mo8-O23	2.032(3)
Mo2-O38	2.306(2)	Mo8-O29	2.359(2)
Mo2-O39	1.834(3)	Mo8-O32	1.863(3)

Mo3-O3	1.686(3)	Mo9-O9	1.682(3)
Mo3-O14	1.824(3)	Mo9-O19	1.820(3)
Mo3-O17	2.023(3)	Mo9-O23	1.824(3)
Mo3-O20	1.834(3)	Mo9-O24	2.038(3)
Mo3-O38	2.367(2)	Mo9-O29	2.349(2)
Mo3-O39	2.010(3)	Mo9-O33	2.019(3)
Mo4-O4	1.685(3)	Mo10-O7	1.829(3)
Mo4-O18	2.003(3)	Mo10-O10	1.696(3)
Mo4-O24	1.797(3)	Mo10-O28	2.333(2)
Mo4-O25	2.047(2)	Mo10-O34	2.000(3)
Mo4-O28	2.359(2)	Mo10-O35	1.824(3)
Mo4-O34	1.853(3)	Mo10-O37	2.036(3)
Mo5-O5	1.688(3)	Mo11-O11	1.687(3)
Mo5-O15	1.812(3)	Mo11-O29	2.330(2)
Mo5-O25	1.824(3)	Mo11-O32	2.055(3)
Mo5-O26	2.027(3)	Mo11-O33	1.839(3)
Mo5-O28	2.349(2)	Mo11-O36	2.020(3)
Mo5-O35	2.046(3)	Mo11-O37	1.816(3)
Mo6-O6	1.685(3)	Mo12-O7	2.023(3)
Mo6-O13	2.009(3)	Mo12-O12	1.679(3)
Mo6-O21	2.017(3)	Mo12-O27	2.335(2)
Mo6-O26	1.815(3)	Mo12-O30	2.031(3)
Mo6-O27	2.339(2)	Mo12-O31	1.843(3)
Mo6-O30	1.851(3)	Mo12-O36	1.821(3)
O6-W3-O5	87.8(5)	O29-W12-O26	72.4(4)
O6-W3-O10	157.3(4)	O35-W12-O26	85.9(4)
O6-W3-O13	73.7(4)	O35-W12-O29	158.0(5)
O11-W3-O6	92.1(5)	O35-W12-O30	90.5(5)
O11-W3-O5	158.5(5)	O35-W12-O36	87.7(5)
O11-W3-O10	85.1(5)	O30-W12-O26	73.3(4)
O11-W3-O13	86.7(4)	O30-W12-O29	86.6(5)
O5-W3-O13	72.7(4)	O30-W12-O36	157.5(5)
O10-W3-O5	86.8(5)	O36-W12-O26	84.2(4)
O10-W3-O13	83.7(4)	O36-W12-O29	86.7(5)
O3-W3-O6	100.2(5)	O38-W12-O26	169.3(5)
O3-W3-O11	102.3(6)	O38-W12-O29	99.0(6)
O3-W3-O5	98.9(6)	O38-W12-O35	103.0(6)
O3-W3-O10	102.4(5)	O38-W12-O30	100.3(6)
O3-W3-O13	169.5(5)	O38-W12-O36	102.0(6)
O4-W2-O8	87.8(5)	O9-W6-O28	84.4(4)
O4-W2-O13	73.3(4)	O9-W6-O22	86.8(5)
O8-W2-O13	84.4(4)	O31-W6-O9	157.8(5)
O9-W2-O4	158.2(5)	O31-W6-O28	73.4(4)
O9-W2-O8	84.7(5)	O31-W6-O22	88.0(5)
O9-W2-O5	91.2(5)	O22-W6-O28	73.2(4)

O9-W2-O13	85.6(4)	O21-W6-O9	86.3(5)
O5-W2-O4	88.4(5)	O21-W6-O31	90.5(5)
O5-W2-O8	158.8(5)	O21-W6-O28	85.1(4)
O5-W2-O13	74.6(4)	O21-W6-O22	157.8(5)
O2-W2-O4	100.3(5)	O16-W6-O9	102.1(6)
O2-W2-O8	102.6(5)	O16-W6-O31	100.1(6)
O2-W2-O9	101.4(5)	O16-W6-O28	170.6(5)
O2-W2-O5	98.6(5)	O16-W6-O22	100.2(6)
O2-W2-O13	170.5(5)	O16-W6-O21	101.9(6)
O4-W1-O6	86.7(5)	O37-W11-O33	87.4(5)
O4-W1-O12	157.1(5)	O37-W11-O27	84.2(4)
O4-W1-O13	72.8(4)	O36-W11-O37	85.9(5)
O6-W1-O13	73.0(4)	O36-W11-O33	157.5(5)
O7-W1-O4	91.7(5)	O36-W11-O27	85.0(4)
O7-W1-O6	157.8(5)	O33-W11-O27	73.0(4)
O7-W1-O12	87.2(5)	O34-W11-O37	158.4(5)
O7-W1-O13	85.4(4)	O34-W11-O36	90.5(5)
O12-W1-O6	85.8(5)	O34-W11-O33	87.9(5)
O12-W1-O13	84.3(4)	O34-W11-O27	74.3(4)
O1-W1-O4	100.6(6)	O40-W11-O37	102.4(6)
O1-W1-O6	99.5(5)	O40-W11-O36	101.1(5)
O1-W1-O7	102.5(5)	O40-W11-O33	101.2(6)
O1-W1-O12	101.9(6)	O40-W11-O34	99.2(6)
O1-W1-O13	170.0(5)	O40-W11-O27	171.2(5)
O8-W5-O26	87.3(4)	O22-W7-O28	73.7(4)
O8-W5-O20	92.9(5)	O10-W7-O28	86.0(4)
O8-W5-O30	158.7(5)	O10-W7-O22	91.7(5)
O8-W5-O21	85.7(5)	O10-W7-O23	85.3(5)
O20-W5-O26	73.2(4)	O10-W7-O32	157.9(5)
O20-W5-O30	87.1(5)	O23-W7-O28	83.2(4)
O20-W5-O21	156.8(5)	O23-W7-O22	156.9(5)
O30-W5-O26	72.3(4)	O32-W7-O28	72.9(4)
O21-W5-O26	83.6(4)	O32-W7-O22	88.4(5)
O21-W5-O30	85.9(5)	O32-W7-O23	85.9(5)
O15-W5-O26	167.9(4)	O17-W7-O28	170.1(5)
O15-W5-O8	103.4(5)	O17-W7-O22	99.5(6)
O15-W5-O20	100.3(6)	O17-W7-O10	101.6(5)
O15-W5-O30	97.6(5)	O17-W7-O23	103.6(6)
O15-W5-O21	102.5(6)	O17-W7-O32	100.1(6)
O37-W10-O31	158.0(5)	O11-W8-O27	84.3(4)
O37-W10-O28	86.7(4)	O33-W8-O11	158.8(5)
O37-W10-O35	85.7(5)	O33-W8-O24	88.6(5)
O37-W10-O32	91.1(5)	O33-W8-O27	74.4(4)
O31-W10-O28	72.2(4)	O23-W8-O11	85.7(5)
O35-W10-O31	86.6(5)	O23-W8-O33	91.1(5)
O35-W10-O28	84.8(4)	O23-W8-O24	156.9(5)
O32-W10-O31	88.0(5)	O23-W8-O27	83.8(5)

O32-W10-O28	72.2(4)	O24-W8-O11	86.3(5)
O32-W10-O35	156.9(5)	O24-W8-O27	73.9(5)
O39-W10-O37	101.8(6)	O18-W8-O11	100.7(6)
O39-W10-O31	99.9(6)	O18-W8-O33	100.5(6)
O39-W10-O28	169.5(5)	O18-W8-O23	103.2(6)
O39-W10-O35	102.0(6)	O18-W8-O24	99.6(6)
O39-W10-O32	101.0(6)	O18-W8-O27	171.6(5)
O7-W4-O26	84.2(4)	O25-W9-O24	157.6(5)
O7-W4-O20	85.7(5)	O25-W9-O34	87.2(5)
O20-W4-O26	72.3(4)	O25-W9-O27	84.5(5)
O25-W4-O26	86.2(5)	O12-W9-O25	86.2(5)
O25-W4-O7	85.4(5)	O12-W9-O24	90.9(5)
O25-W4-O20	157.5(5)	O12-W9-O34	157.3(5)
O29-W4-O26	73.4(4)	O12-W9-O27	85.2(4)
O29-W4-O7	157.6(5)	O24-W9-O34	87.0(5)
O29-W4-O20	87.4(5)	O24-W9-O27	73.1(4)
O29-W4-O25	93.1(5)	O34-W9-O27	72.6(4)
O14-W4-O26	171.0(5)	O19-W9-O25	101.9(6)
O14-W4-O7	101.1(5)	O19-W9-O12	102.3(5)
O14-W4-O20	100.6(6)	O19-W9-O24	100.4(6)
O14-W4-O25	101.4(6)	O19-W9-O34	100.3(6)
O14-W4-O29	101.1(5)	O19-W9-O27	170.4(5)
O28-Si1-O26	109.5(5)	O27-Si1-O26	109.3(5)
O13-Si1-O26	109.4(5)	O27-Si1-O28	109.8(6)
O13-Si1-O28	108.5(5)	O27-Si1-O13	110.3(6)
W1-O4-W2	122.4(6)	W2-O13-W1	91.5(3)
W12-O26-W5	92.7(4)	Si1-O13-W3	123.6(5)
W12-O26-W4	91.7(3)	Si1-O13-W2	126.0(6)
W4-O26-W5	91.6(4)	Si1-O13-W1	123.1(5)
Si1-O26-W12	124.2(5)	W8-O27-W11	90.4(4)
Si1-O26-W5	123.3(5)	W8-O27-W9	90.9(4)
Si1-O26-W4	124.1(5)	W9-O27-W11	90.9(3)
W10-O37-W11	151.6(7)	Si1-O27-W11	124.7(6)
W5-O8-W2	151.9(7)	Si1-O27-W8	125.2(5)
W3-O6-W1	122.2(5)	Si1-O27-W9	124.3(6)
W2-O9-W6	150.9(6)	W3-O13-W1	91.1(4)
W1-O7-W4	150.7(7)	W2-O13-W3	91.6(4)
W3-O11-W8	150.9(6)	W12-O35-W10	150.1(6)
W6-O31-W10	122.4(6)	W9-O12-W1	150.4(7)
W5-O20-W4	122.9(6)	W7-O22-W6	120.6(6)
W6-O28-W10	92.0(3)	W7-O10-W3	151.7(6)
W7-O28-W6	92.5(3)	W12-O30-W5	121.8(6)
W7-O28-W10	91.9(3)	W11-O36-W12	150.4(6)
Si1-O28-W6	124.3(5)	W8-O33-W11	122.1(6)
Si1-O28-W10	123.1(5)	W8-O23-W7	153.0(7)
Si1-O28-W7	123.8(5)	W8-O24-W9	122.1(6)
W2-O5-W3	121.1(6)	W11-O34-W9	122.2(6)

W4-O25-W9	151.9(7)	W6-O21-W5	151.0(7)
W4-O29-W12	122.5(6)	W10-O32-W7	123.0(7)
**2**			
W3-O6	1.915(11)	W10-O37	1.902(11)
W3-O11	1.892(12)	W10-O31	1.953(12)
W3-O5	1.968(12)	W10-O28	2.358(10)
W3-O10	1.931(11)	W10-O35	1.940(12)
W3-O13	2.362(10)	W10-O32	1.917(12)
W3-O3	1.687(11)	W10-O39	1.696(12)
W2-O4	1.938(11)	W4-O26	2.346(10)
W2-O8	1.947(11)	W4-O7	1.922(11)
W2-O9	1.880(12)	W4-O20	1.947(12)
W2-O5	1.897(11)	W4-O25	1.894(11)
W2-O13	2.331(10)	W4-O29	1.889(11)
W2-O2	1.706(11)	W4-O14	1.698(12)
W1-O4	1.908(12)	W7-O28	2.331(9)
W1-O6	1.945(11)	W7-O22	1.935(12)
W1-O7	1.886(11)	W7-O10	1.883(11)
W1-O12	1.921(12)	W7-O23	1.923(12)
W1-O13	2.373(10)	W7-O32	1.919(12)
W1-O1	1.686(11)	W7-O17	1.690(12)
W12-O26	2.345(9)	W8-O11	1.928(12)
W12-O29	1.951(12)	W8-O33	1.893(12)
W12-O35	1.872(12)	W8-O23	1.866(12)
W12-O30	1.918(13)	W8-O24	1.917(11)
W12-O36	1.923(12)	W8-O27	2.344(10)
W12-O38	1.700(12)	W8-O18	1.723(12)
W6-O9	1.946(11)	W9-O25	1.908(11)
W6-O31	1.902(11)	W9-O12	1.878(12)
W6-O28	2.340(10)	W9-O24	1.924(12)
W6-O22	1.951(12)	W9-O34	1.985(12)
W6-O21	1.896(12)	W9-O27	2.373(11)
W6-O16	1.689(12)	W9-O19	1.678(12)
W11-O37	1.917(11)	W5-O26	2.352(10)
W11-O36	1.892(11)	W5-O8	1.867(11)
W11-O33	1.935(11)	W5-O20	1.886(12)
W11-O34	1.880(13)	W5-O30	1.971(12)
W11-O27	2.376(10)	W5-O21	1.918(12)
W11-O40	1.685(11)	W5-O15	1.698(12)
Si1-O26	1.641(10)	Si1-O13	1.626(10)
Si1-O28	1.639(10)	Si1-O27	1.604(11)
O6-W3-O5	87.8(5)	O29-W12-O26	72.4(4)
O6-W3-O10	157.3(4)	O35-W12-O26	85.9(4)

O6-W3-O13	73.7(4)	O35-W12-O29	158.0(5)
O11-W3-O6	92.1(5)	O35-W12-O30	90.5(5)
O11-W3-O5	158.5(5)	O35-W12-O36	87.7(5)
O11-W3-O10	85.1(5)	O30-W12-O26	73.3(4)
O11-W3-O13	86.7(4)	O30-W12-O29	86.6(5)
O5-W3-O13	72.7(4)	O30-W12-O36	157.5(5)
O10-W3-O5	86.8(5)	O36-W12-O26	84.2(4)
O10-W3-O13	83.7(4)	O36-W12-O29	86.7(5)
O3-W3-O6	100.2(5)	O38-W12-O26	169.3(5)
O3-W3-O11	102.3(6)	O38-W12-O29	99.0(6)
O3-W3-O5	98.9(6)	O38-W12-O35	103.0(6)
O3-W3-O10	102.4(5)	O38-W12-O30	100.3(6)
O3-W3-O13	169.5(5)	O38-W12-O36	102.0(6)
O4-W2-O8	87.8(5)	O9-W6-O28	84.4(4)
O4-W2-O13	73.3(4)	O9-W6-O22	86.8(5)
O8-W2-O13	84.4(4)	O31-W6-O9	157.8(5)
O9-W2-O4	158.2(5)	O31-W6-O28	73.4(4)
O9-W2-O8	84.7(5)	O31-W6-O22	88.0(5)
O9-W2-O5	91.2(5)	O22-W6-O28	73.2(4)
O9-W2-O13	85.6(4)	O21-W6-O9	86.3(5)
O5-W2-O4	88.4(5)	O21-W6-O31	90.5(5)
O5-W2-O8	158.8(5)	O21-W6-O28	85.1(4)
O5-W2-O13	74.6(4)	O21-W6-O22	157.8(5)
O2-W2-O4	100.3(5)	O16-W6-O9	102.1(6)
O2-W2-O8	102.6(5)	O16-W6-O31	100.1(6)
O2-W2-O9	101.4(5)	O16-W6-O28	170.6(5)
O2-W2-O5	98.6(5)	O16-W6-O22	100.2(6)
O2-W2-O13	170.5(5)	O16-W6-O21	101.9(6)
O4-W1-O6	86.7(5)	O37-W11-O33	87.4(5)
O4-W1-O12	157.1(5)	O37-W11-O27	84.2(4)
O4-W1-O13	72.8(4)	O36-W11-O37	85.9(5)
O6-W1-O13	73.0(4)	O36-W11-O33	157.5(5)
O7-W1-O4	91.7(5)	O36-W11-O27	85.0(4)
O7-W1-O6	157.8(5)	O33-W11-O27	73.0(4)
O7-W1-O12	87.2(5)	O34-W11-O37	158.4(5)
O7-W1-O13	85.4(4)	O34-W11-O36	90.5(5)
O12-W1-O6	85.8(5)	O34-W11-O33	87.9(5)
O12-W1-O13	84.3(4)	O34-W11-O27	74.3(4)
O1-W1-O4	100.6(6)	O40-W11-O37	102.4(6)
O1-W1-O6	99.5(5)	O40-W11-O36	101.1(5)
O1-W1-O7	102.5(5)	O40-W11-O33	101.2(6)
O1-W1-O12	101.9(6)	O40-W11-O34	99.2(6)
O1-W1-O13	170.0(5)	O40-W11-O27	171.2(5)
O8-W5-O26	87.3(4)	O37-W10-O31	158.0(5)
O8-W5-O20	92.9(5)	O37-W10-O28	86.7(4)
O8-W5-O30	158.7(5)	O37-W10-O35	85.7(5)
O8-W5-O21	85.7(5)	O37-W10-O32	91.1(5)

O20-W5-O26	73.2(4)	O31-W10-O28	72.2(4)
O20-W5-O30	87.1(5)	O35-W10-O31	86.6(5)
O20-W5-O21	156.8(5)	O35-W10-O28	84.8(4)
O30-W5-O26	72.3(4)	O32-W10-O31	88.0(5)
O21-W5-O26	83.6(4)	O32-W10-O28	72.2(4)
O21-W5-O30	85.9(5)	O32-W10-O35	156.9(5)
O15-W5-O26	167.9(4)	O39-W10-O37	101.8(6)
O15-W5-O8	103.4(5)	O39-W10-O31	99.9(6)
O15-W5-O20	100.3(6)	O39-W10-O28	169.5(5)
O15-W5-O30	97.6(5)	O39-W10-O35	102.0(6)
O15-W5-O21	102.5(6)	O39-W10-O32	101.0(6)
O7-W4-O26	84.2(4)	O22-W7-O28	73.7(4)
O7-W4-O20	85.7(5)	O10-W7-O28	86.0(4)
O20-W4-O26	72.3(4)	O10-W7-O22	91.7(5)
O25-W4-O26	86.2(5)	O10-W7-O23	85.3(5)
O25-W4-O7	85.4(5)	O10-W7-O32	157.9(5)
O25-W4-O20	157.5(5)	O23-W7-O28	83.2(4)
O29-W4-O26	73.4(4)	O23-W7-O22	156.9(5)
O29-W4-O7	157.6(5)	O32-W7-O28	72.9(4)
O29-W4-O20	87.4(5)	O32-W7-O22	88.4(5)
O29-W4-O25	93.1(5)	O32-W7-O23	85.9(5)
O14-W4-O26	171.0(5)	O17-W7-O28	170.1(5)
O14-W4-O7	101.1(5)	O17-W7-O22	99.5(6)
O14-W4-O20	100.6(6)	O17-W7-O10	101.6(5)
O14-W4-O25	101.4(6)	O17-W7-O23	103.6(6)
O14-W4-O29	101.1(5)	O17-W7-O32	100.1(6)
O25-W9-O24	157.6(5)	O11-W8-O27	84.3(4)
O25-W9-O34	87.2(5)	O33-W8-O11	158.8(5)
O25-W9-O27	84.5(5)	O33-W8-O24	88.6(5)
O12-W9-O25	86.2(5)	O33-W8-O27	74.4(4)
O12-W9-O24	90.9(5)	O23-W8-O11	85.7(5)
O12-W9-O34	157.3(5)	O23-W8-O33	91.1(5)
O12-W9-O27	85.2(4)	O23-W8-O24	156.9(5)
O24-W9-O34	87.0(5)	O23-W8-O27	83.8(5)
O24-W9-O27	73.1(4)	O24-W8-O11	86.3(5)
O34-W9-O27	72.6(4)	O24-W8-O27	73.9(5)
O19-W9-O25	101.9(6)	O18-W8-O11	100.7(6)
O19-W9-O12	102.3(5)	O18-W8-O33	100.5(6)
O19-W9-O24	100.4(6)	O18-W8-O23	103.2(6)
O19-W9-O34	100.3(6)	O18-W8-O24	99.6(6)
O19-W9-O27	170.4(5)	O18-W8-O27	171.6(5)
O28-Si1-O26	109.5(5)	W8-O33-W11	122.1(6)
O13-Si1-O26	109.4(5)	W8-O23-W7	153.0(7)
O13-Si1-O28	108.5(5)	W8-O24-W9	122.1(6)
O27-Si1-O26	109.3(5)	W11-O34-W9	122.2(6)
O27-Si1-O28	109.8(6)	W6-O21-W5	151.0(7)
O27-Si1-O13	110.3(6)	W10-O32-W7	123.0(7)

W1-O4-W2	122.4(6)	W3-O13-W1	91.1(4)
W12-O26-W5	92.7(4)	W2-O13-W3	91.6(4)
W12-O26-W4	91.7(3)	W2-O13-W1	91.5(3)
W4-O26-W5	91.6(4)	Si1-O13-W3	123.6(5)
Si1-O26-W12	124.2(5)	Si1-O13-W2	126.0(6)
Si1-O26-W5	123.3(5)	Si1-O13-W1	123.1(5)
Si1-O26-W4	124.1(5)	W8-O27-W11	90.4(4)
W10-O37-W11	151.6(7)	W8-O27-W9	90.9(4)
W5-O8-W2	151.9(7)	W9-O27-W11	90.9(3)
W3-O6-W1	122.2(5)	Si1-O27-W11	124.7(6)
W2-O9-W6	150.9(6)	Si1-O27-W8	125.2(5)
W1-O7-W4	150.7(7)	Si1-O27-W9	124.3(6)
W3-O11-W8	150.9(6)	W2-O5-W3	121.1(6)
W6-O31-W10	122.4(6)	W4-O25-W9	151.9(7)
W5-O20-W4	122.9(6)	W4-O29-W12	122.5(6)
W6-O28-W10	92.0(3)	W12-O35-W10	150.1(6)
W7-O28-W6	92.5(3)	W9-O12-W1	150.4(7)
W7-O28-W10	91.9(3)	W7-O22-W6	120.6(6)
Si1-O28-W6	124.3(5)	W7-O10-W3	151.7(6)
Si1-O28-W10	123.1(5)	W12-O30-W5	121.8(6)
Si1-O28-W7	123.8(5)	W11-O36-W12	150.4(6)
**3**			
P1-O77	1.537(5)	P2-O37	1.532(5)
P1-O78	1.542(5)	P2-O38	1.538(5)
P1-O79	1.529(5)	P2-O39	1.528(5)
P1-O80	1.528(5)	P2-O40	1.523(5)
Mo1-O1	1.670(5)	Mo2-O2	1.677(5)
Mo1-O4	1.867(5)	Mo2-O5	1.889(5)
Mo1-O5	1.944(5)	Mo2-O6	1.905(5)
Mo1-O7	1.904(5)	Mo2-O9	1.917(5)
Mo1-O8	1.952(5)	Mo2-O10	1.947(5)
Mo1-O37	2.452(5)	Mo2-O39	2.428(5)
Mo3-O3	1.673(5)	Mo4-O8	1.946(5)
Mo3-O4	1.938(5)	Mo4-O14	1.674(5)
Mo3-O6	1.906(5)	Mo4-O19	1.907(6)
Mo3-O11	1.913(5)	Mo4-O20	1.937(6)
Mo3-O12	1.925(5)	Mo4-O26	1.893(5)
Mo3-O40	2.436(5)	Mo4-O37	2.428(5)
Mo5-O9	1.938(5)	Mo6-O10	1.896(5)
Mo5-O15	1.678(6)	Mo6-O16	1.690(5)
Mo5-O20	1.905(5)	Mo6-O21	1.934(5)
Mo5-O21	1.932(5)	Mo6-O22	1.895(5)
Mo5-O27	1.879(5)	Mo6-O28	1.911(5)
Mo5-O39	2.435(4)	Mo6-O39	2.440(4)
Mo7-O11	1.914(5)	Mo8-O12	1.908(5)
Mo7-O17	1.689(5)	Mo8-O18	1.678(6)
Mo7-O22	1.914(5)	Mo8-O23	1.972(6)

Mo7-O23	1.901(6)	Mo8-O24	1.872(6)
Mo7-O29	1.925(5)	Mo8-O30	1.922(5)
Mo7-O40	2.449(4)	Mo8-O40	2.444(4)
Mo9-O7	1.932(5)	Mo10-O26	1.919(5)
Mo9-O13	1.663(5)	Mo10-O27	1.958(5)
Mo9-O19	1.930(6)	Mo10-O31	1.888(5)
Mo9-O24	1.934(6)	Mo10-O32	1.924(5)
Mo9-O25	1.921(5)	Mo10-O35	1.681(5)
Mo9-O37	2.447(5)	Mo10-O38	2.431(5)
Mo11-O28	1.912(5)	Mo12-O25	1.877(5)
Mo11-O29	1.916(5)	Mo12-O30	1.889(5)
Mo11-O32	1.919(5)	Mo12-O31	1.927(5)
Mo11-O33	1.893(5)	Mo12-O33	1.953(5)
Mo11-O36	1.682(5)	Mo12-O34	1.676(5)
Mo11-O38	2.428(4)	Mo12-O38	2.440(4)
Mo13-O41	1.684(6)	Mo14-O42	1.686(5)
Mo13-O44	1.858(6)	Mo14-O45	1.903(5)
Mo13-O45	1.926(5)	Mo14-O46	1.967(5)
Mo13-O47	1.910(6)	Mo14-O49	1.883(5)
Mo13-O48	1.980(6)	Mo14-O50	1.903(5)
Mo13-O77	2.427(5)	Mo14-O79	2.423(4)
Mo15-O43	1.679(6)	Mo16-O47	1.947(6)
Mo15-O44	1.970(6)	Mo16-O53	1.944(5)
Mo15-O46	1.858(5)	Mo16-O58	1.894(5)
Mo15-O51	1.855(6)	Mo16-O59	1.677(6)
Mo15-O52	1.985(5)	Mo16-O70	1.872(5)
Mo15-O78	2.429(5)	Mo16-O77	2.433(5)
Mo17-O48	1.883(6)	Mo18-O49	1.966(5)
Mo17-O54	1.864(5)	Mo18-O54	1.962(5)
Mo17-O58	1.948(5)	Mo18-O55	1.860(5)
Mo17-O60	1.682(6)	Mo18-O61	1.683(5)
Mo17-O65	1.965(6)	Mo18-O66	1.867(5)
Mo17-O77	2.438(5)	Mo18-O79	2.437(4)
Mo19-O50	1.911(5)	Mo20-O51	1.980(6)
Mo19-O55	1.988(5)	Mo20-O56	1.950(5)
Mo19-O56	1.857(5)	Mo20-O57	1.889(5)
Mo19-O62	1.681(5)	Mo20-O63	1.670(6)
Mo19-O67	1.933(5)	Mo20-O68	1.887(6)
Mo19-O79	2.443(5)	Mo20-O78	2.438(5)
Mo21-O52	1.915(5)	Mo22-O65	1.847(6)
Mo21-O53	1.887(5)	Mo22-O66	1.965(5)
Mo21-O57	1.943(6)	Mo22-O71	1.867(6)
Mo21-O64	1.683(5)	Mo22-O72	1.948(6)
Mo21-O69	1.914(5)	Mo22-O75	1.702(5)
Mo21-O78	2.440(5)	Mo22-O80	2.438(5)
Mo23-O69	1.894(5)	Mo24-O67	1.866(5)
Mo23-O70	1.944(5)	Mo24-O68	1.955(6)

Mo23-O71	1.966(5)	Mo24-O72	1.892(6)
Mo23-O73	1.880(5)	Mo24-O73	1.958(6)
Mo23-O74	1.685(6)	Mo24-O76	1.673(6)
Mo23-O80	2.442(5)	Mo24-O80	2.446(5)
O77-P1-O78	109.1(3)	O3-Mo3-O4	101.8(3)
O79-P1-O77	109.1(3)	O3-Mo3-O6	104.0(2)
O79-P1-O78	110.1(3)	O3-Mo3-O11	100.6(3)
O80-P1-O77	108.9(3)	O3-Mo3-O12	99.8(2)
O80-P1-O78	109.3(3)	O3-Mo3-O40	170.3(2)
O80-P1-O79	110.3(3)	O4-Mo3-O40	84.40(18)
O37-P2-O38	109.4(3)	O6-Mo3-O4	85.0(2)
O39-P2-O37	109.5(3)	O6-Mo3-O11	90.1(2)
O39-P2-O38	109.5(3)	O6-Mo3-O12	156.0(2)
O40-P2-O37	109.8(3)	O6-Mo3-O40	83.86(19)
O40-P2-O38	109.2(3)	O11-Mo3-O4	157.7(2)
O40-P2-O39	109.4(3)	O11-Mo3-O12	87.5(2)
O1-Mo1-O4	104.7(3)	O11-Mo3-O40	73.39(18)
O1-Mo1-O5	102.8(3)	O12-Mo3-O4	88.2(2)
O1-Mo1-O7	100.9(3)	O12-Mo3-O40	72.60(19)
O1-Mo1-O8	98.6(2)	O8-Mo4-O37	72.54(18)
O1-Mo1-O37	168.6(2)	O14-Mo4-O8	99.4(3)
O4-Mo1-O5	86.2(2)	O14-Mo4-O19	100.5(3)
O4-Mo1-O7	91.6(2)	O14-Mo4-O20	101.9(3)
O4-Mo1-O8	156.5(2)	O14-Mo4-O26	104.3(3)
O4-Mo1-O37	85.34(19)	O14-Mo4-O37	169.9(2)
O5-Mo1-O8	85.3(2)	O19-Mo4-O8	87.6(2)
O5-Mo1-O37	83.11(18)	O19-Mo4-O20	157.4(2)
O7-Mo1-O5	156.0(2)	O19-Mo4-O37	73.60(19)
O7-Mo1-O8	87.4(2)	O20-Mo4-O8	86.2(2)
O7-Mo1-O37	72.90(18)	O20-Mo4-O37	83.81(19)
O8-Mo1-O37	71.88(18)	O26-Mo4-O8	156.2(2)
O2-Mo2-O5	103.0(3)	O26-Mo4-O19	90.4(2)
O2-Mo2-O6	101.7(3)	O26-Mo4-O20	86.6(2)
O2-Mo2-O9	101.8(2)	O26-Mo4-O37	84.19(19)
O2-Mo2-O10	100.7(3)	O9-Mo5-O39	72.27(18)
O2-Mo2-O39	170.7(2)	O15-Mo5-O9	100.2(3)
O5-Mo2-O6	86.3(2)	O15-Mo5-O20	102.2(3)
O5-Mo2-O9	89.7(2)	O15-Mo5-O21	101.2(3)
O5-Mo2-O10	156.3(2)	O15-Mo5-O27	102.6(3)
O5-Mo2-O39	84.66(19)	O15-Mo5-O39	170.4(2)
O6-Mo2-O9	156.5(2)	O20-Mo5-O9	86.2(2)
O6-Mo2-O10	88.1(2)	O20-Mo5-O21	156.4(2)
O6-Mo2-O39	83.86(19)	O20-Mo5-O39	83.53(19)
O9-Mo2-O10	86.3(2)	O21-Mo5-O9	87.0(2)
O9-Mo2-O39	72.75(18)	O21-Mo5-O39	72.86(19)
O10-Mo2-O39	71.80(18)	O27-Mo5-O9	157.1(2)
O10-Mo6-O21	86.9(2)	O27-Mo5-O20	88.3(2)

O10-Mo6-O28	156.0(2)	O27-Mo5-O21	89.3(2)
O10-Mo6-O39	72.30(18)	O27-Mo5-O39	85.07(18)
O16-Mo6-O10	103.2(3)	O30-Mo8-O23	85.8(2)
O16-Mo6-O21	100.9(3)	O30-Mo8-O40	82.83(19)
O16-Mo6-O22	102.7(3)	O7-Mo9-O24	88.2(2)
O16-Mo6-O28	100.8(3)	O7-Mo9-O37	72.59(18)
O16-Mo6-O39	172.1(2)	O13-Mo9-O7	102.0(3)
O21-Mo6-O39	72.71(19)	O13-Mo9-O19	101.9(3)
O22-Mo6-O10	89.9(2)	O13-Mo9-O24	102.1(3)
O22-Mo6-O21	156.3(2)	O13-Mo9-O25	102.1(3)
O22-Mo6-O28	85.8(2)	O13-Mo9-O37	172.4(3)
O22-Mo6-O39	83.96(19)	O19-Mo9-O7	86.7(2)
O28-Mo6-O21	87.6(2)	O19-Mo9-O24	156.0(2)
O28-Mo6-O39	83.78(19)	O19-Mo9-O37	72.80(19)
O11-Mo7-O22	88.4(2)	O24-Mo9-O37	83.33(19)
O11-Mo7-O29	156.9(2)	O25-Mo9-O7	155.7(2)
O11-Mo7-O40	73.08(18)	O25-Mo9-O19	90.5(2)
O17-Mo7-O11	101.9(3)	O25-Mo9-O24	84.6(2)
O17-Mo7-O22	103.3(3)	O25-Mo9-O37	83.55(19)
O17-Mo7-O23	101.1(3)	O26-Mo10-O27	83.4(2)
O17-Mo7-O29	101.1(3)	O26-Mo10-O32	156.1(2)
O17-Mo7-O40	172.4(3)	O26-Mo10-O38	83.90(18)
O22-Mo7-O29	84.0(2)	O27-Mo10-O38	83.67(18)
O22-Mo7-O40	82.48(18)	O31-Mo10-O26	91.0(2)
O23-Mo7-O11	88.4(2)	O31-Mo10-O27	156.4(2)
O23-Mo7-O22	155.5(2)	O31-Mo10-O32	88.8(2)
O23-Mo7-O29	89.5(2)	O31-Mo10-O38	72.91(18)
O23-Mo7-O40	73.4(2)	O32-Mo10-O27	87.3(2)
O29-Mo7-O40	84.26(19)	O32-Mo10-O38	73.23(18)
O12-Mo8-O23	86.6(2)	O35-Mo10-O26	102.9(2)
O12-Mo8-O30	155.5(2)	O35-Mo10-O27	101.3(2)
O12-Mo8-O40	72.67(19)	O35-Mo10-O31	102.3(2)
O18-Mo8-O12	103.0(3)	O35-Mo10-O32	100.4(2)
O18-Mo8-O23	98.9(3)	O35-Mo10-O38	171.9(2)
O18-Mo8-O24	103.9(3)	O28-Mo11-O29	84.9(2)
O18-Mo8-O30	101.2(3)	O28-Mo11-O32	87.9(2)
O18-Mo8-O40	170.3(3)	O28-Mo11-O38	83.60(19)
O23-Mo8-O40	72.38(19)	O29-Mo11-O32	157.7(2)
O24-Mo8-O12	90.5(2)	O29-Mo11-O38	84.87(19)
O24-Mo8-O23	157.1(2)	O32-Mo11-O38	73.37(18)
O24-Mo8-O30	87.5(2)	O33-Mo11-O28	156.7(2)
O24-Mo8-O40	85.0(2)	O33-Mo11-O29	89.9(2)
O36-Mo11-O28	101.4(2)	O33-Mo11-O32	88.5(2)
O36-Mo11-O29	101.9(3)	O33-Mo11-O38	73.26(18)
O36-Mo11-O32	100.2(2)	O58-Mo17-O65	83.6(2)
O36-Mo11-O33	102.0(2)	O58-Mo17-O77	71.73(19)
O36-Mo11-O38	171.8(2)	O60-Mo17-O48	102.3(3)

O25-Mo12-O30	87.7(2)	O60-Mo17-O54	102.7(3)
O25-Mo12-O31	89.1(2)	O60-Mo17-O58	101.3(3)
O25-Mo12-O33	156.2(2)	O60-Mo17-O65	101.5(3)
O25-Mo12-O38	84.19(19)	O60-Mo17-O77	172.1(2)
O30-Mo12-O31	155.5(2)	O65-Mo17-O77	81.77(19)
O30-Mo12-O33	88.0(2)	O49-Mo18-O79	72.28(18)
O30-Mo12-O38	83.4(2)	O54-Mo18-O49	83.8(2)
O31-Mo12-O33	85.3(2)	O54-Mo18-O79	82.36(18)
O31-Mo12-O38	72.11(18)	O55-Mo18-O49	87.7(2)
O33-Mo12-O38	72.06(18)	O55-Mo18-O54	155.5(2)
O34-Mo12-O25	103.6(3)	O55-Mo18-O66	93.8(2)
O34-Mo12-O30	102.4(3)	O55-Mo18-O79	73.19(19)
O34-Mo12-O31	102.0(3)	O61-Mo18-O49	99.8(3)
O34-Mo12-O33	100.2(2)	O61-Mo18-O54	101.2(3)
O34-Mo12-O38	170.3(2)	O61-Mo18-O55	102.8(3)
O41-Mo13-O44	102.7(3)	O61-Mo18-O66	102.6(3)
O41-Mo13-O45	101.9(3)	O61-Mo18-O79	171.0(2)
O41-Mo13-O47	101.7(3)	O66-Mo18-O49	156.6(2)
O41-Mo13-O48	99.8(3)	O66-Mo18-O54	85.3(2)
O41-Mo13-O77	170.9(3)	O66-Mo18-O79	85.82(19)
O44-Mo13-O45	87.8(2)	O50-Mo19-O55	85.2(2)
O44-Mo13-O47	91.5(2)	O50-Mo19-O67	154.8(2)
O44-Mo13-O48	157.4(2)	O50-Mo19-O79	72.35(18)
O44-Mo13-O77	84.8(2)	O55-Mo19-O79	71.11(18)
O45-Mo13-O48	85.7(2)	O56-Mo19-O50	92.3(2)
O45-Mo13-O77	83.3(2)	O56-Mo19-O55	155.2(2)
O47-Mo13-O45	155.9(2)	O56-Mo19-O67	87.3(2)
O47-Mo13-O48	85.8(2)	O56-Mo19-O79	84.61(19)
O47-Mo13-O77	72.7(2)	O62-Mo19-O50	102.1(2)
O48-Mo13-O77	72.97(19)	O62-Mo19-O55	101.7(2)
O42-Mo14-O45	101.9(2)	O62-Mo19-O56	103.0(2)
O42-Mo14-O46	101.1(2)	O62-Mo19-O67	102.5(3)
O42-Mo14-O49	102.4(2)	O62-Mo19-O79	171.0(2)
O42-Mo14-O50	101.1(2)	O67-Mo19-O55	84.7(2)
O42-Mo14-O79	172.8(2)	O67-Mo19-O79	82.55(19)
O45-Mo14-O46	82.6(2)	O51-Mo20-O78	71.68(19)
O45-Mo14-O79	84.4(2)	O56-Mo20-O51	84.8(2)
O46-Mo14-O79	82.89(18)	O56-Mo20-O78	81.89(18)
O49-Mo14-O45	91.0(2)	O57-Mo20-O51	86.4(2)
O49-Mo14-O46	156.4(2)	O57-Mo20-O56	155.2(2)
O49-Mo14-O50	90.1(2)	O57-Mo20-O78	73.3(2)
O49-Mo14-O79	73.90(19)	O63-Mo20-O51	100.9(3)
O50-Mo14-O45	156.1(2)	O63-Mo20-O56	101.1(3)
O50-Mo14-O46	87.0(2)	O63-Mo20-O57	103.4(3)
O50-Mo14-O79	72.95(18)	O63-Mo20-O68	103.1(3)
O43-Mo15-O44	101.3(3)	O63-Mo20-O78	171.9(3)
O43-Mo15-O46	103.6(3)	O68-Mo20-O51	155.3(2)

O43-Mo15-O51	102.3(3)	O68-Mo20-O56	85.3(2)
O43-Mo15-O52	98.8(3)	O68-Mo20-O57	93.3(2)
O43-Mo15-O78	170.1(2)	O68-Mo20-O78	84.6(2)
O44-Mo15-O52	83.0(2)	O52-Mo21-O57	87.1(3)
O44-Mo15-O78	82.0(2)	O52-Mo21-O78	72.99(19)
O46-Mo15-O44	85.2(2)	O53-Mo21-O52	90.0(2)
O46-Mo15-O52	156.3(2)	O53-Mo21-O57	156.5(2)
O46-Mo15-O78	85.90(19)	O53-Mo21-O69	86.6(2)
O51-Mo15-O44	155.7(2)	O53-Mo21-O78	84.43(19)
O51-Mo15-O46	95.1(2)	O57-Mo21-O78	72.42(19)
O51-Mo15-O52	87.4(2)	O64-Mo21-O52	99.7(3)
O51-Mo15-O78	73.8(2)	O64-Mo21-O53	102.9(3)
O52-Mo15-O78	72.16(18)	O64-Mo21-O57	100.6(3)
O47-Mo16-O77	71.96(19)	O64-Mo21-O69	103.7(3)
O53-Mo16-O47	86.1(2)	O64-Mo21-O78	169.8(2)
O53-Mo16-O77	83.33(19)	O69-Mo21-O52	156.6(2)
O58-Mo16-O47	86.8(2)	O69-Mo21-O57	86.8(2)
O58-Mo16-O53	156.0(2)	O69-Mo21-O78	83.60(19)
O58-Mo16-O77	72.69(19)	O65-Mo22-O66	85.4(2)
O59-Mo16-O47	100.5(3)	O65-Mo22-O71	93.9(2)
O59-Mo16-O53	102.3(3)	O65-Mo22-O72	155.6(2)
O59-Mo16-O58	101.5(3)	O65-Mo22-O80	84.6(2)
O59-Mo16-O70	101.7(3)	O66-Mo22-O80	82.99(19)
O59-Mo16-O77	170.4(2)	O71-Mo22-O66	156.7(2)
O70-Mo16-O47	157.5(2)	O71-Mo22-O72	87.4(2)
O70-Mo16-O53	85.8(2)	O71-Mo22-O80	73.7(2)
O70-Mo16-O58	92.3(2)	O72-Mo22-O66	84.0(2)
O70-Mo16-O77	86.3(2)	O72-Mo22-O80	72.43(19)
O48-Mo17-O58	89.0(2)	O75-Mo22-O65	102.9(3)
O48-Mo17-O65	156.0(2)	O75-Mo22-O66	100.3(2)
O48-Mo17-O77	74.2(2)	O75-Mo22-O71	102.6(3)
O54-Mo17-O48	93.2(2)	O75-Mo22-O72	100.6(3)
O54-Mo17-O58	154.9(2)	O75-Mo22-O80	172.0(2)
O54-Mo17-O65	84.3(2)	O69-Mo23-O70	84.8(2)
O54-Mo17-O77	84.76(19)	O69-Mo23-O71	156.3(2)
O70-Mo23-O71	86.2(2)	O69-Mo23-O80	85.03(19)
O70-Mo23-O80	83.00(19)	O67-Mo24-O68	85.7(2)
O71-Mo23-O80	72.13(19)	O67-Mo24-O72	92.7(2)
O73-Mo23-O69	92.7(2)	O67-Mo24-O73	155.4(2)
O73-Mo23-O70	155.6(2)	O67-Mo24-O80	85.0(2)
O73-Mo23-O71	86.6(2)	O68-Mo24-O73	84.6(2)
O73-Mo23-O80	72.6(2)	O68-Mo24-O80	82.16(19)
O74-Mo23-O69	101.7(3)	O76-Mo24-O67	102.8(3)
O74-Mo23-O70	103.1(3)	O76-Mo24-O68	103.0(3)
O74-Mo23-O71	101.6(3)	O76-Mo24-O72	101.5(3)
O74-Mo23-O73	101.2(3)	O76-Mo24-O73	101.4(3)
O74-Mo23-O80	171.2(2)	O76-Mo24-O80	170.8(2)

O72-Mo24-O68	155.2(2)	O72-Mo24-O80	73.1(2)
O72-Mo24-O73	86.9(2)	O73-Mo24-O80	71.32(19)

Table S2. Selected hydrogen bonds of compounds **1-3.**

**1**				
D-H...A	D-H (Å)	H...A (Å)	D...A (Å)	D-H...A(°)
O1W-H1WA...O30iii	0.85	2.16	2.965(4)	159
O1W-H1WB...O10vi	0.85	2.25	2.989(5)	145
N5-H5A...O8v	0.86	2.22	3.053(5)	162
N6-H6...O1Wviii	0.86	2.19	3.042(7)	173
N7-H7A...O42ix	0.86	2.15	2.849(6)	138
N7-H7A...O10x	0.86	2.58	3.056(4)	116
N7-H7A...O37x	0.86	2.57	3.212(5)	132
N8-H8A...O32vi	0.86	1.87	2.710(5)	165
O41-H41...O1Wiv	0.82	1.91	2.729(4)	173
O43-H43...O2vii	0.82	1.94	2.680(6)	150
C3-H3...O12x	0.93	2.53	3.179(5)	128
C5-H5...O5ii	0.93	2.4	3.302(7)	164
C8-H8...O4	0.93	2.6	3.410(6)	146
C9-H9...O42xi	0.93	2.42	3.069(7)	127
C10-H10...O6ii	0.93	2.59	3.260(6)	129
C11-H11...O2ii	0.93	2.56	3.156(6)	123
C11-H11...O15ii	0.93	2.39	3.295(6)	164
C13-H13...O10x	0.93	2.45	3.002(5)	118
C20-H20...O43i	0.93	2.58	3.469(5)	160
C21-H21...O2iv	0.93	2.4	3.292(7)	160
C23-H23...O9i	0.93	2.36	3.176(6)	146
C24-H24...O1v	0.93	2.34	3.003(8)	128
C24-H24...O14v	0.93	2.54	3.211(7)	129
C25-H25...O11vi	0.93	2.54	3.235(7)	132
C26-H26...O40iv	0.93	2.51	3.380(6)	156
**2**				
N1-H1...O20ii	0.88	1.92	2.77(3)	162
O1W-H1WB...O6viii	0.87	2.32	2.972(18)	132
N4-H4...O19iii	0.88	2.54	3.04(2)	117
N4-H4...O41iii	0.88	2.14	2.87(2)	139
N6-H6...O41vii	0.88	2.37	3.04(3)	134

N9-H9...O4vi	0.88	2.56	3.18(3)	128
O42-H42...O1Wv	0.84	1.86	2.701(19)	176
O44-H44...O17v	0.84	2.09	2.68(2)	127
C2-H2...O15i	0.95	2.11	3.04(2)	166
C7-H7...O44	0.95	2.54	3.44(2)	158
C8-H8...O39	0.95	2.41	3.22(2)	143
C10-H10...O17v	0.95	2.40	3.31(3)	162
C11-H11...O31i	0.95	2.40	3.18(4)	139
C11-H11...O38i	0.95	2.42	3.1(3)	129
C12-H12...O14ii	0.95	2.56	3.26(3)	131
C13-H13...O2v	0.95	2.46	3.39(3)	167
C18-H18...O18iv	0.95	2.43	3.36(3)	165
C20-H20...O1iii	0.95	2.49	3.18(2)	129
C21-H21...O40	0.95	2.55	3.35(3)	142
C22-H22...O1Wvi	0.95	2.12	3.05(3)	167
C22-H22...O3vi	0.95	2.54	3.02(3)	112
C24-H24...O19iii	0.95	2.53	3.04(2)	114
C25-H25...O23iv	0.95	2.46	3.38(3)	163
**3**				
O1W-H1WA...O18	0.87	2.42	2.962(9)	121
O1W-H1WA...O2Wiii	0.87	2.50	2.789(9)	100
N2-H2...O8ii	0.88	1.82	2.683(9)	167
O1W-H1WB...O2v	0.87	2.34	3.020(10)	135
O1W-H1WB...O5W	0.87	2.29	2.891(8)	126
O2W-H2WA...O1Wiii	0.87	2.35	2.789(9)	112
O2W-H2WA...N2ii	0.87	2.50	3.208(12)	139
N4-H4...O9v	0.88	2.51	3.147(10)	130
N4-H4...O20v	0.88	2.33	3.181(10)	164
O2W-H2WB...O1Wvi	0.87	1.86	2.638(11)	148
N6-H6...O42iv	0.88	2.56	3.046(10)	115
N6-H6...O87	0.88	2.17	2.910(11)	141
O3W-H3WB...O21	0.87	2.49	2.996(8)	118
O3W-H3WB...O27	0.87	2.30	3.158(8)	168
O4W-H4WA...O48iv	0.87	2.35	3.043(8)	137
N8-H8A...O81vii	0.88	2.26	3.015(12)	144
O5W-H5WA...O23	0.87	2.14	2.915(8)	149
O5W-H5WB...O1W	0.87	2.36	2.891(8)	120
N12-H12A...O3Wiv	0.88	2.15	2.999(10)	160
N14Z-H14Z...O44vi	0.88	2.56	3.406(12)	161
N14Z-H14Z...O47vi	0.88	2.47	3.052(12)	124
O82-H82...O4W	0.84	1.93	2.753(8)	168
O84-H84...O16iv	0.84	2.21	2.917(10)	142
O86-H86...O75v	0.84	1.95	2.679(10)	145
O88-H88...O3W	0.84	1.93	2.772(8)	174
C3-H3...O83	0.95	2.51	3.332(10)	145
C5-H5...O3iii	0.95	2.44	3.363(9)	165
C8-H8...O12iii	0.95	2.19	3.137(11)	172

C13-H13...O17iv	0.95	2.36	3.104(13)	135
C13-H13...O83	0.95	2.59	3.360(11)	138
C16-H16...O41iv	0.95	2.49	3.111(10)	123
C18-H18...O61v	0.95	2.5	3.438(11)	170
C20-H20...O15v	0.95	2.57	3.265(11)	131
C21-H21...O66v	0.95	2.39	3.288(11)	157
C23-H23...O35	0.95	2.55	3.424(10)	153
C23-H23...O42iv	0.95	2.46	3.006(10)	116
C24-H24...O60v	0.95	2.51	3.189(12)	128
C25-H25...O4Wvii	0.95	2.3	3.160(13)	150
C29-H29...O41iv	0.95	2.52	3.313(11)	141
C31-H31...O36iv	0.95	2.32	3.264(11)	172
C33-H33...O15v	0.95	2.47	3.299(11)	146
C34-H34...O35v	0.95	2.55	3.084(10)	116
C35-H35...O26v	0.95	2.51	3.137(10)	123
C35-H35...O81v	0.95	2.48	3.161(11)	129
C36-H36...O29iv	0.95	2.34	3.209(10)	153
C37-H37...O62viii	0.95	2.41	3.227(11)	144
C38-H38...O87iv	0.95	2.48	3.027(12)	117
C39-H39...O28iv	0.95	2.51	3.376(13)	152
C42-H42...O86i	0.95	2.58	3.449(10)	152
C44-H44...O74ix	0.95	2.59	3.477(10)	155
C47-H47...O63iv	0.95	2.49	3.290(12)	142
C48-H48...O64ix	0.95	2.26	3.180(11)	163
C49-H49...O75	0.95	2.38	3.277(14)	157
C50-H50...O73ix	0.95	2.27	3.136(16)	151
C50-H50...O76ix	0.95	2.27	3.177(17)	123
C52-H52...O59	0.95	2.56	3.499(11)	171

Symmetry codes for compound **1**: (i) 1-x,1-y,1-z; (ii) 1-x,-y,2-z; (iii) 1-x,1-y,2-z; (iv) -x,1-y,1-z; (v) x,1+y,z; (vi) -1+x,y,z; (vii) 1+x,y,z; (viii) x,-1+y,z; (ix) 1+x,y,1+z; (x) 2-x,1-y,2-z; (xi) -x,-y,1-z

Symmetry codes for compound **2** : (i) 1-x,1-y,-z; (ii) 2-x,2-y,-z; (iii) 2-x,2-y,-z; (iv) 1-x,1-y,1-z; (v) 1+x,y,z; (vi) x,-1+y,z; (vii) -1+x,-1+y,z; (viii) 1-x,2-y,1-z

Symmetry codes for compound **3** : (i) x,1+y,z; (ii) -x,2-y,-z; (iii) 1-x,1-y,-z; (iv) -1+x,y,z; (v) x,-1+y,z; (vi) -1+x,1+y,z; (vii) 1+x,-1+y,z; (viii) -1+x,-1+y,z; (ix) 1-x,2-y,1-z
